# Proteomics and Transcriptomics of BJAB Cells Expressing the Epstein-Barr Virus Noncoding RNAs EBER1 and EBER2

**DOI:** 10.1371/journal.pone.0124638

**Published:** 2015-06-29

**Authors:** Genaro Pimienta, Victor Fok, Maria Haslip, Maria Nagy, Seyedtaghi Takyar, Joan A Steitz

**Affiliations:** 1 Department of Molecular Biophysics and Biochemistry, Howard Hughes Medical Institute, Yale University School of Medicine, New Haven, Connecticut, United States of America; 2 Section of Pulmonary, Critical Care, and Sleep Medicine, Department of Internal Medicine, Yale University School of Medicine, New Haven, Connecticut, United States of America; 3 Department of Genetics, Howard Hughes Medical Institute, Yale University School of Medicine, New Haven, Connecticut, United States of America; University of Nebraska—Lincoln, UNITED STATES

## Abstract

In Epstein-Barr virus (EBV) latent infection, the EBV-encoded RNAs EBER1 and EBER2 accumulate in the host cell nucleus to ~10^6^ copies. While the expression of EBERs in cell lines is associated with transformation, a mechanistic explanation of their roles in EBV latency remains elusive. To identify EBER-specific gene expression features, we compared the proteome and mRNA transcriptome from BJAB cells (an EBV-negative B lymphoma cell line) stably transfected with an empty plasmid or with one carrying both EBER genes. We identified ~1800 proteins with at least 2 SILAC pair measurements, of which only 8 and 12 were up- and downregulated ≥ 2-fold, respectively. One upregulated protein was PIK3AP1, a B-cell specific protein adapter known to activate the PI3K-AKT signaling pathway, which regulates alternative splicing and translation in addition to its pro-survival effects. In the mRNA-seq data, the mRNA levels for some of the proteins changing in the SILAC data did not change. We instead observed isoform switch events. We validated the most relevant findings with biochemical assays. These corroborated the upregulation of PIK3AP1 and AKT activation in BJAB cells expressing high levels of both EBERs and EBNA1 (a surrogate of Burkitt’s lymphoma EBV latency I) relative to those expressing only EBNA1. The mRNA-seq data in these cells showed multiple upregulated oncogenes whose mRNAs are enriched for 3´-UTR AU-rich elements (AREs), such as *ccl3*, *ccr7*, *il10*, *vegfa* and *zeb1*. The CCL3, CCR7, IL10 and VEGFA proteins promote cell proliferation and are associated with EBV-mediated lymphomas. In EBV latency, ZEB1 represses the transcription of ZEBRA, an EBV lytic phase activation factor. We previously found that EBER1 interacts with AUF1 *in vivo* and proposed stabilization of ARE-containing mRNAs. Thus, the ~10^6^ copies of EBER1 may promote not only cell proliferation due to an increase in the levels of ARE-containing genes like *ccl3*, *ccr7*, *il10*, and *vegfa*, but also the maintenance of latency, through higher levels of *zeb1*.

## Introduction

EBV is a human γ-herpesvirus that infects primarily B cells, establishing a life-long latent infection that is innocuous in most healthy individuals; but in those with immunodeficiency, infection can cause cancer [1, 2]. The EBV episome contains about 90 open reading frames, but only 9 proteins (EBNA1, EBNA2, EBNA3A, EBNA3B, EBNA3C, EBNALP, LMP1, LMP2A and LMP2B) are expressed during viral latency [1, 2]. EBV latency comprises three well-investigated stages (I, II and III), plus the less-characterized latency 0 [3]. Each stage has a unique viral transcriptional program and thus produces a different subset of EBV-encoded proteins [3]. In addition to the latent viral protein EBNA1, the EBV noncoding (nc) RNAs, EBER1 and EBER2, are expressed at high levels in all four latency stages [1, 4].

The EBERs are transcribed by RNA Polymerase III, lack a poly-A tail and were first described in EBV-infected B cells as the RNA components of ribonucleoprotein (RNP) complexes that include the cellular autoantigen La [5, 6]. Their nucleotide sequences are ~50% identical and their experimentally-defined secondary structures contain multiple stems and loops [7]. The EBERs reside in the nucleus, where they associate with at least two proteins, La and L22 [8]. The RNA chaperone La binds both EBERs, whereas the ribosomal protein L22 associates only with EBER1 [8, 9]. Recently, we have shown using stable isotope labeling of amino acids in cell culture (SILAC) proteomics that EBER1 also interacts specifically with various hnRNPs, including the AU-rich element (ARE)-binding protein AUF1 (hnRNP D) [10].

Two cell lines (AKATA and BJAB) that derive from Burkitt’s lymphoma human tumors, which at the time of isolation were EBV-infected [11, 12], are available. When expressed alone in these two cell lines, the EBERs can induce cell proliferation associated with a tumorigenic phenotype both *in vitro* and in an *in vivo* mouse assay [11–14]. Furthermore, the infection of AKATA cells with EBER1/2-minus EBV bacmids has shown that the EBERs, and EBER2 in particular, contribute to an EBV-mediated transformation phenotype [15]. These results have been challenged by studies showing that the EBV-mediated transformation of freshly isolated lymphocytes is not compromised by the knockdown of the EBERs [16, 17]. An explanation for this lack of EBER-deletion effects in freshly-isolated lymphocytes may be that the EBER-specific oncogenic functions are dependent on a biochemical environment that primes tumorigenesis, like that in AKATA cells. In AKATA cells, the expression of EBERs at physiologically-relevant levels induces a reduced tumorigenic effect in mice, compared to cells infected by EBV [14], suggesting that the EBERs may have functions redundant with those of the EBV-encoded proteins produced during latency [1, 2, 18].

To identify unique EBER-mediated gene expression features, we integrated the EBER1 and EBER2 genes into a single chromosomal locus in BJAB cells (an EBV-negative B lymphoma cell line) and compared the proteome and mRNA transcriptome of these BJAB-EBER1/2 cells to a BJAB-CTL counterpart carrying an empty vector at the same site.

The SILAC results comparing BJAB-EBER1/2 and BJAB-CTL reveal that only a tiny fraction of the proteins identified are up- or downregulated ≥ 2-fold; in some cases, the mRNA levels do not correlate, suggesting post-transcriptional regulation of these genes. To corroborate these results, we also analyzed mRNA-seq data of BJAB cells co-expressing higher amounts of EBNA1 and EBER1/2 (BJAB-EBNA1-EBER1/2 cells) versus EBNA1 alone (BJAB-EBNA1 cells).

## Materials and Methods

### FRT strategy

Stable BJAB (American Type Culture Collection, ATCC) Flp-In cell lines were created using a Flippase (Flp) recognition target site-directed recombination system (Invitrogen). We inserted a recombination plasmid either empty (control) or containing the EcoRI-J fragment from EBV that encodes the EBERs [19]. We next selected the FRT-containing cells by expanding them in increasing amounts of hygromycin (50–250 μg/ml).

### pCEP4 vector constructs

To introduce more than one copy of the EcoRI-J fragment into the pCEP4 plasmid (Invitrogen), we took advantage of the BglII and HindIII restriction sites up- and downstream, respectively, of the CMV promoter and of a BamHI one downstream the HindIII site as described in S1 File. To make stable cell lines, we transfected BJAB cells with each of these plasmids by electroporating at 230 V with 10 μg of pCEP4 empty or containing the EcoRI-J fragment, plus a similar amount of a GFP-expressing plasmid (pGFP-MAX) to trace the transfection efficiency as described above before [19]. We next selected the pCEP4-containing cells by expanding them in increasing amounts of hygromycin (50–250 μg/ml).

### SILAC proteomics sample preparation

We optimized a proteomics protocol that allows us to identify more than a thousand proteins (S1A Fig). To identify effects of only EBER1/2 expression on the cellular proteome, the FRT-derived cell lines BJAB-CTL and BJAB-EBER1/2 were cultured 10 doubling-times in their respective SILAC media to reach saturation-labeling (more than 95%). BJAB-CTL cells were labeled with light isotopes, ^12^C_6_-,^14^N_4_-Arg and ^12^C_6_-,^14^N_4_-Lysine (Arg0/Lys0), and BJAB-EBER1/2 cells with heavy labels, ^13^C_6_-,^14^N_4_-Arg and ^13^C_6_-,^14^N_4_-Lysine (Arg6/Lys6), using RPMI-1640 media especially formulated for SILAC experiments (Pierce), supplemented with a penicillin/streptomycin cocktail 10,000 units/ml (Gibco), plus 50 μg/ml hygromicin (Calbiochem). After labeling and large-scale cell expansion, equal numbers of the BJAB-CTL (Arg0/Lys0) and BJAB-EBER1/2 (Arg6/Lys6) cells were pooled to produce about 50 mg (total cell pellet weight) of the SILAC mixture.

To increase the number of proteins identified, the SILAC mixture was divided in two and the following process performed twice. First, the complexity of the SILAC mixture at the protein level was reduced using a subcellular fractionation kit (Pierce) that partitioned the sample into 4 compartments (membrane/pellet, cytoplasm, nuclear and chromatin-bound), to which protease inhibitors were added–one tablet of EDTA-free protease inhibitor cocktail (Sigma) per 50 ml of lysis solution. Each fraction was in turn precipitated overnight at -20°C with pre-chilled acetone. The protein pellet from each subcellular fraction was next dissolved in 2 ml of Invitrosol (Invitrogen), a mass spectrometry (MS) compatible surfactant. To dissolve the pellet completely, we vortexed and sonicated each sample vigorously. The Invitrosol-dissolved, fully denatured proteins were next reduced with fresh 10 mM DTT (Sigma) and their free cysteines alkylated with 100 mM iodoacetamide (Sigma), followed by overnight digestion with sequence grade Trypsin (Promega).

After trypsin digestion of each subcellular fraction, the tryptic peptide mixtures were desalted with a Sep-Pak C18 solid phase extraction cartridge (Waters), dried in a vacuum concentrator and coupled to batch-wise strong cation exchange (SCX) that entailed 6 pH elution steps. For this, each sample was loaded onto 0.1 mg of PolySULFOETHYL A, 3 μm, 300 Å (PolyLC), previously resuspended and pre-equilibrated in citric acid 50 mM; 2.5% v/v acetonitrile; pH approximately 1.5. After a 2-hour incubation and continuous mixing, the bound material was applied to a microcentrifuge TopTip (Glygen) and washed twice with equilibration buffer by centrifugation at no more than 8000 rpm. The bound peptides were next eluted according to their charge, with a step-wise increase in the pH of the equilibration buffer at pH 3, 4, 5, 6 and 8. All 48 elution samples were desalted with Clean-up C18 tips and dried in a vacuum concentrator.

Samples were submitted to W. M. Keck Foundation proteomics facility at Yale University where three technical replicates each of the 48 peptide fractions were further separated by reversed-phase liquid chromatography (RP-LC), coupled online to an LTQ-Orbitrap mass spectrometer. For data analysis, we used the software MaxQuant-Andromeda, specially developed to interpret SILAC proteomics data collected on LTQ-Orbitrap instruments [20].

To discard technical inconsistencies in SILAC labeling and/or data collection, we confirmed that the total number of SILAC ratios fit approximately within a Gaussian curve (S1B Fig). We next determined that the signal-to-noise total ion intensity of the peptides measured in the up- and downregulated SILAC ratios was not close to background levels, ruling out potential artifacts (S1C Fig). We also show a representative fragmentation pattern of ADAD2, FCRLA/1, EIF2B4 and PIK3AP1 (S1D Fig).

### RNA sample preparation for mRNA-seq and qRT-PCR experiments

RNA samples were extracted from confluent cells (BJAB or 293T from American Type Culture Collection, ATCC) as indicated in the main text with Tryzol (Invitrogen), followed by isopropanol precipitation and removal of salts with 70% v/v ethanol/H_2_O. We further removed traces of genomic DNA from the samples by DNase treatment with an RNA cleanup kit RNeasy (Qiagen). After quality inspection on an agarose gel and 260/280 absorbance, samples were immediately flash-frozen and stored in 5–10 μl aliquots until use.

### First-strand cDNA synthesis

For qRT-PCR assays, we used a SuperScript III First-Strand Synthesis System (Invitrogen) and followed the manufacturer’s protocol. In all cases, the RNA used was treated with DNase as described above. To validate gene-specific total mRNA levels calculated in the bioinformatics analysis, we used the FastStart Universal SYBR Green Master (ROX) reagent (Roche). We collected the melting curves on a StepOne Plus Real-Time PCR System (Applied Biosystems) and analyzed the data on the instrument’s software version 2.2.2.

### Northern blots

Northern blots to detect both EBERs and fluorescence in situ hybridization (FISH) assays specific for EBER1 were performed as described previously [8].

### Western blots

Total cell lysates were prepared with an NP40 lysis buffer. Specific antibodies were used as indicated, and a species-compatible horseradish peroxidase (HRP) coupled secondary antibodies and the signal developed with SuperSignal West Femto Maximum Sensitivity Substrate (Thermo). Antibody recognition signal bands were captured on a G:BOX SYNGENE scanner. Band intensities were calculated with ImageJ.

### Cell counting

Each BJAB cell line used in this study was diluted to about 10^6^ cells per ml and allowed to proliferate for 72 hours. Cells were counted every 24 hours and the average values of three biological replicates presented.

## Results

### Comparison of proteomics and transcriptomics data from BJAB cells stably expressing EBER1 and EBER2

We stably expressed EBER1 and EBER2 in BJAB cells using two strategies–the Flp-In genome integration strategy (Invitrogen) and the pCEP4 episomal vector (Invitrogen). In both cases, we introduced the EcoRI-J fragment from the EBV genome, which encodes the two EBERs and contains upstream motifs shown previously to enhance their transcription [6, 19].

In the first case, homologous recombination Flp-In reagents were used to integrate a single flippase recognition target (FRT) site into the BJAB genome, followed by the recombination of either an empty expression plasmid or one containing the EcoRI-J fragment mentioned above into the FRT site. After hygromycin selection we obtained the BJAB-CTL and BJAB-EBER1/2 cell lines, respectively. In our hands, BJAB cells stop proliferating when diluted to below 10^6^ cells per ml. Due to this difficulty, we selected a population of FRT-containing cells by expanding them in increasing amounts of hygromycin 50–250 μg/ml.

Alternatively, we ligated into the pCEP4 vector (a plasmid that encodes the EBV protein EBNA1 for stabilization) 4 tandem copies (4X) of the EcoRI-J fragment. We electroporated BJAB cells with the pCEP4 vector, empty or containing the 4X EcoRI-J fragment, and after hygromycin selection obtained the BJAB-EBNA1 (control) and BJAB-EBNA1-EBER1/2 cell lines, respectively.

**Fig 1:**
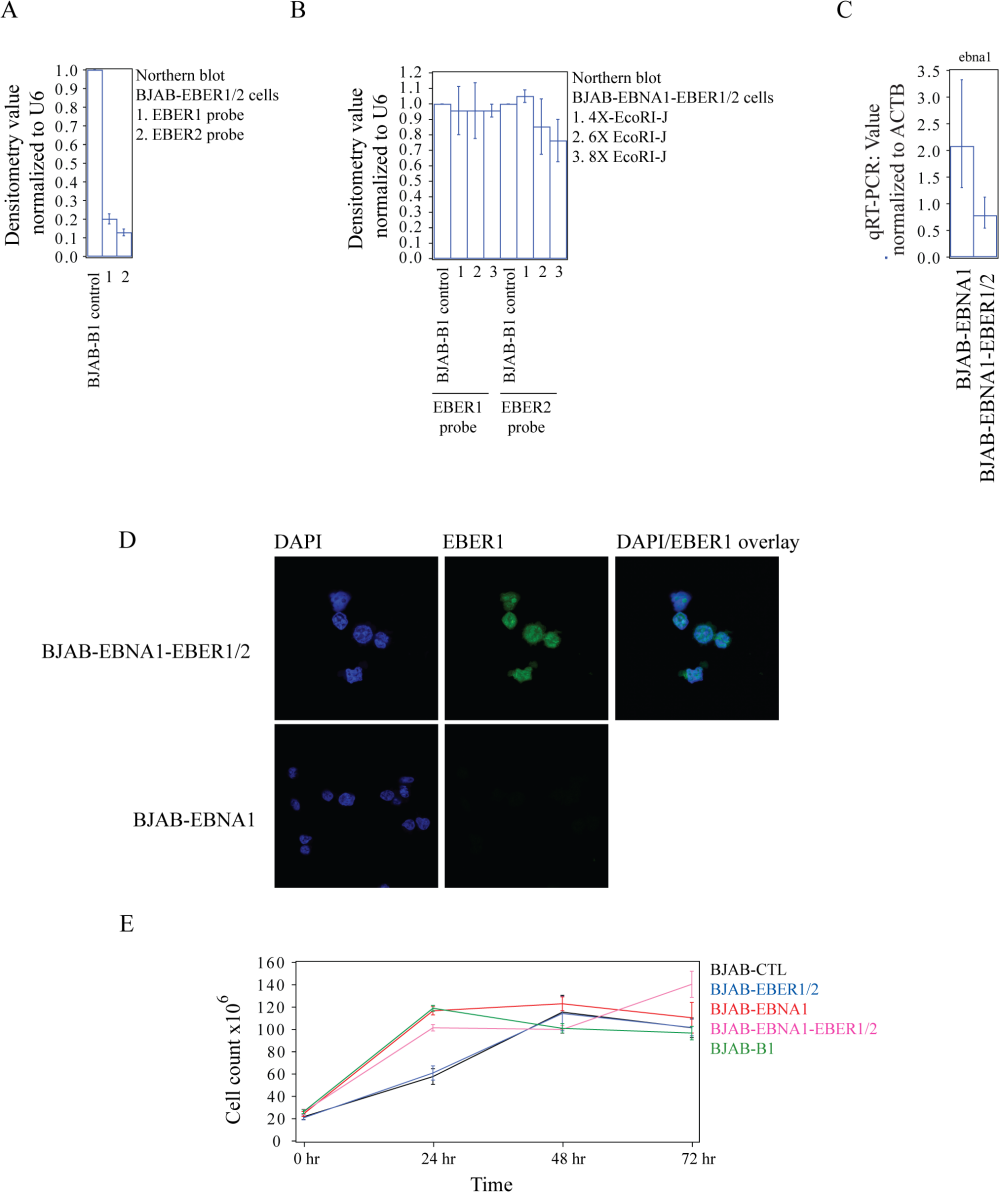
Expression levels of EBER1 and EBER2 in BJAB cells. **(A)** In BJAB-EBER1/2 cells (FRT), EBER1 and EBER2 are expressed to about one tenth of their levels found in EBV-infected BJAB cells (BJAB-B1) set to 1. The densitometry values are the average of three biological replicates. **(B)** BJAB cells stably transfected with the ppCEP4 vector containing four (4X), six (6X) or eight (8X) copies of the EcoRI-J fragment expressed similar levels of EBER1 and EBER2, compared to BJAB-B1 cells. The densitometry values are the average of three biological replicates. **(C)** qRT-PCR assays specific for *ebna1* mRNA in BJAB-EBNA1 and BJAB-EBNA1-EBER1/2, compared to BJAB-B1 cells. **(D)** FISH image shows the consistent expression of EBER1 in the nucleus of BJAB-EBNA1-EBER1/2 cells. In **(A-C)** the values are normalized to actin (ACTB) as a control and the error bars reflect the standard deviation from three biological replicates. **(E**) Proliferation assays of BJAB cell lines. Cells were counted in triplicate every 24 hours.

We confirmed the expression of EBER1 and EBER2 in both cell systems by northern blot (NB) and compared their levels with those of BJAB-B1 cells, which are EBV-infected BJAB cells (Fig 1A and 1B and S2A and S2B Fig). In the FRT system, the amounts of EBER1 and EBER2 were approximately 10-fold lower than in BJAB-B1 cells (Fig 1A). In the pCEP4 system, the levels of EBER1 and EBER2 were comparable to those of BJAB-B1 cells (Fig 1B). We also confirmed by qRT-PCR that the expression of *ebna1* mRNA was not substantially different in BJAB-EBNA1 and BJAB-EBNA1-EBER1/2 (Fig 1C). In BJAB-EBNA1-EBER1/2 cells, the expression of EBER1 appeared consistent from cell to cell, as determined by fluorescence *in situ* hybridization (FISH) analyses (Fig 1D). Finally, we confirmed the previously reported effect of high-level EBER expression in BJAB cells on the proliferation rate by counting cells over time [11]. We found that the activation of BJAB cells by either high-level EBER expression (BJAB-EBNA1-EBER1/2), EBNA1 alone (BJAB-EBNA1) or EBV infection (BJAB-B1), increases the proliferation rate of these cells, when compared to the parental BJAB control (Fig 1E). BJAB cells expressing low levels of EBER1 and EBER2 (BJAB-EBER1/2) did not show an increase in the proliferation rate (Fig 1E).

To discover EBER-specific effects on protein levels, we performed a comparative study of BJAB-EBER1/2 and BJAB-CTL cells. A comprehensive whole-cell proteomics experiment entails identifying at least 1000 proteins with annotated functions spanning a large repertoire of gene ontology (GO) terms [20]. To achieve this goal, we employed a protein and peptide fractionation scheme coupled to SILAC quantitative proteomics and high-resolution mass spectrometry (S1A Fig). For proteomics data analysis, we used the software MaxQuant-Andromeda [21] with default parameters and the latest International Protein Index (IPI) fasta database [22], based on high-quality gene models, including those from Reference Sequence (RefSeq). We identified a total of 1990 (≥ 1 SILAC counts) or 1820 (≥ 2 SILAC counts) individual protein names, based on their unique gene names. The SILAC values were expressed as the ratio of BJAB-EBER1/2 to BJAB-CTL (S2 File). When the software identified more than one protein with identical peptides or non-unique gene names due to poor annotation, these proteins were sorted into protein groups [21]. This way, the 1990 protein IDs corresponded to 1802 protein groups with at least one SILAC count (S2 File). An example is the FCRLA/FCRL1 protein group with a significant fold-change discussed below.

**Fig 2:**
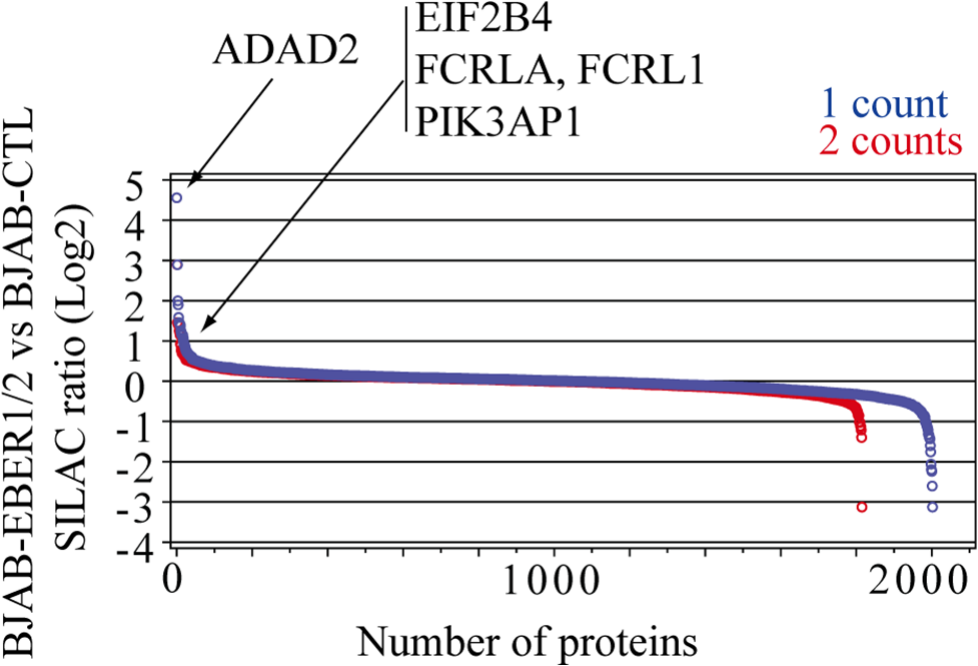
SILAC ratios of the proteins identified. Log2 SILAC ratios (BJAB-EBER1/2 *vs* BJAB-CTL) plotted against the number of proteins identified. Ratios with ≥ 1 SILAC pair count are colored blue and those with ≥ 2 counts red. Representative proteins upregulated are indicated.

Changes in the levels of most proteins were subtle (Fig 2). In the list of individual proteins with at least 2 SILAC counts, we found 8 upregulated and 12 downregulated ≥ 2-fold (Table 1). All of the up- and downregulated proteins had good posterior error probability (PEP) scores (S2 File). We also confirmed that their tandem mass spectrometry (MS/MS) fragmentation spectra were of good quality–the spectra had a good signal-to-noise ratio and most amino acids in the peptide sequence were explained by the MS/MS data (S1B, S1C and S1D Fig).

**Table 1:** BJAB-EBER1/2 *vs* BJAB-CTL calculated ratios.

Gene symbol UNIPROT entry	SILAC Proteomics ≥ 2 counts	mRNA-seq 1st biological replicate	mRNA-seq2nd biological replicate
**ADAD2**	**7.40**	**-**	**-**
**Q8NCV1**			
**NDUFS2**	**2.40**	**0.76**	**0.98**
**O75306**			
**UBE2C**	**2.73**	**0.97**	**0.82**
**O00762**			
FCRLA*	**2.65**	**2.90**	**1.18**
**Q7L513**			
FCRL1*	**2.65**	**1.97**	**1.60**
**Q96LA6**			
**EIF2B4**	**2.60**	**1.00**	**1.00**
**Q9UI10**			
**TRIT1**	**2.37**	**0.81**	**0.93**
**I6L9K0**			
**GOLIM4**	**2.31**	**1.92**	**1.72**
**O00461**			
**PIK3AP1**	**2.21**	**1.70**	**1.76**
**Q6ZUJ8**			
**BCAT1**	**0.49**	**-**	**1.37**
**P54687**			
**ATP5L**	**0.49**	**0.95**	**0.93**
**O75964**			
**PROX1**	**0.49**	**3.20**	**2.0**
**Q92786**			
**CDKN2AIP**	**0.45**	**1.20**	**1.25**
**Q96HQ2**			
**RBM19**	**0.43**	**0.87**	**0.88**
**Q9Y4C8**			
**RAI1**	**0.37**	**0.99**	**1.15**
**Q7Z5J4**			
**SRPUL**	**0.11**	**-**	**-**
**O60687**			
**NUP93**	**0.08**	**1.06**	**1.04**
**Q8N1F7**			
**NXNL2**	**0.04**	**-**	**-**
**Q5VZ03**			
**STRBP**	**0.03**	**1.53**	**1.47**
**Q96SI9**			
**LRRC14B**	**0.01**	**1.36**	**1.32**
**A6NHZ5**			

* FCRLA and FCRL1 were identified as a protein group by MaxQuant-Andromeda

**Fig 3:**
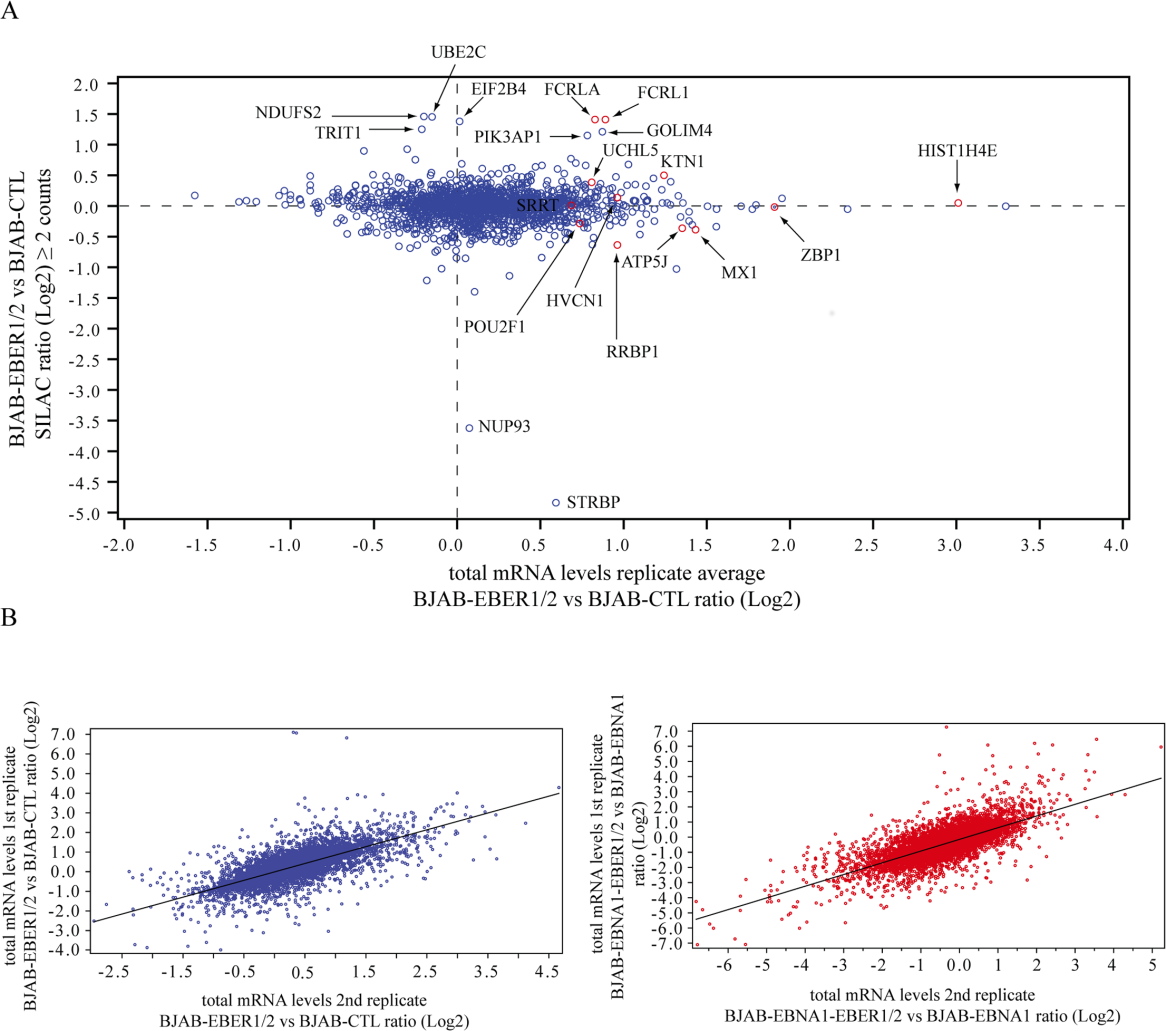
SILAC proteomics and mRNA-seq transcriptomics. **(A)** Cross-correlation of SILAC ratios (≥ 2 counts) and their corresponding total mRNA levels. The y-axis shows the Log2 values of the SILAC EBER/CTL ratio. The x-axis indicates for each SILAC ratio its corresponding Log2 total mRNA levels obtained by mRNA-seq. The mRNA values were averaged from the two biological replicates collected. Proteins with a significant SILAC ratio are indicated. Those with a significant fold-change in the mRNA-seq data are colored red. **(B)** Plots showing the cross-correlation between fold-changes (Log2 values) in the two biological replicates of each comparison: BJAB-EBER1/2 *vs* BJAB-CTL comparison (blue), BJAB-EBNA1-EBER1/2 *vs* BJAB-EBNA2 comparison (red).

We next compared the protein levels with at least 2 SILAC counts to the average values of their corresponding total mRNA abundances quantified in two mRNA-seq biological replicates of BJAB-EBER1/2 and BJAB-CTL cells (S2 File). Plotting the SILAC ratios and their corresponding mRNA-seq gene levels revealed that, in some cases, there was no correlation between the protein and total mRNA fold-changes (Fig 3A and Table 1). With the exception of FCRLA and FCRL1 found in the SILAC data, some of the significant fold-changes at the total mRNA level (red dots) had corresponding SILAC values close to 1 (Fig 3A). Likewise, EIF2B4 was upregulated at the protein level, but not in the mRNA-seq data (blue dots) (Fig 3A). These discrepancies between protein and total mRNA levels suggest post-transcriptional regulation, which cannot be assessed by standard proteomics experiments.

To calculate total mRNA levels and isoform switch events, we used the Tuxedo bioinformatics suite (TopHat, Cufflinks and Cuffdiff tools) [23] as described in S1 File. To assure that the statistical metrics were calculated by Cuffdiff with high confidence, especially at the isoform level, we collected in the two datasets (BJAB-EBER1/2 *vs* BJAB-CTL and BJAB-EBNA1-EBER1/2 *vs* BJAB-EBNA1 comparisons) about 200 and 100 million paired-end reads of 76bp, in the first and second biological replicates, respectively. While the two replicates in each comparison showed a high correlation in fold-change values (Fig 3B), the extremely high coverage of more than 200 million paired-end reads resulted in a larger number of genes with a significant q-value (statistically-relevant up- or downregulation fold-change), despite their similar fold-changes in the two replicates per comparison (S3 File). Accordingly, we based the GO analysis and discussion of the mRNA-seq data below on the first biological replicate of each comparison (BJAB-EBER1/2 *vs* BJAB-CTL and BJAB-EBNA1-EBER1/2 *vs* BJAB-EBNA1). The fold-changes in the second biological replicate are reported in S3 File.

In the first biological replicate of the BJAB-EBER1/2 *vs* BJAB-CTL comparison, Cuffdiff computed the total mRNA levels of 13,853 genes with statistical confidence and–mirroring the few changes at the protein level–determined that only 207 genes (1.5%) were upregulated and 33 (0.2%) downregulated with significant q-values (S4 File). Many of the proteins encoded by the genes up- or downregulated in the mRNA-seq data were not identified in the proteomics experiment (S2 File), most probably due to their low intracellular abundance and/or trypsin digestion pattern.

In many cases, a change not apparent at the total mRNA level was masked by isoform-switch events, due to either alternative splicing or promoter usage (S3 File). At the isoform level, Cuffdiff calculated the levels of 23,178 alternative transcripts and determined that 1557 (6.7%) had a significant fold-change, according to their q-value (S5 File). In the pool of significantly changing isoforms, 379 (1.6%) appeared to have been derived from a significant alternative splicing change (S6 File) and another 461 (1.9%) transcripts from significant altered promoter usage (S7 File).

### Biological functions and validation of proteins upregulated in the SILAC profile

**Fig 4:**
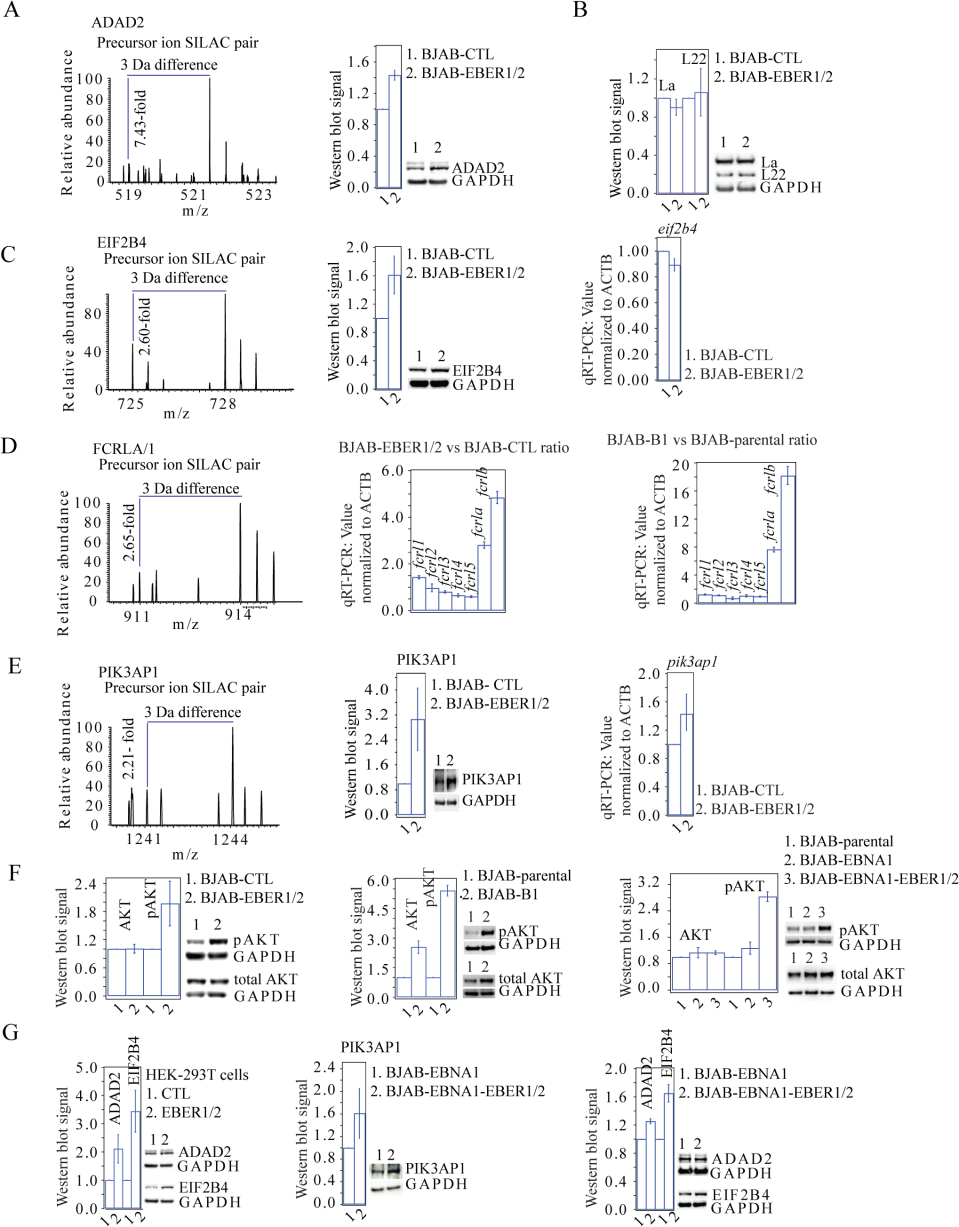
WB and qRT-PCR validation assays. **(A)** Representative precursor ion SILAC pair MS spectra and WB assay for ADAD2 in the BJAB-EBER1/2 *vs* BJAB-CTL comparison. **(B)** WB assays showing the measured fold-changes for La and L22 in the BJAB-EBER1/2 *vs* BJAB-CTL comparison, which in our SILAC data do not change. **(C)** Representative precursor ion SILAC pair MS spectra for EIF2B4, plus the corresponding WB and qRT-PCR assays in the BJAB-EBER1/2 *vs* BJAB-CTL comparison. **(D)** Representative precursor ion SILAC pair MS spectra and WB assay for the FCRLA/1 protein group in the BJAB-EBER1/2 *vs* BJAB-CTL comparison. Total mRNA level fold-changes based on qRT-PCR assays for the family of FCRLs expressed in BJAB cells in the two indicated comparisons. **(E)** Representative precursor ion SILAC pair MS spectra for PIK3AP1, plus the corresponding WB and qRT-PCR assays in the BJAB-EBER1/2 *vs* BJAB-CTL comparison. **(F)** WB assays for total AKT and pAKT in the three indicated comparisons. In all panels, the WB densitometry and qRT-PCR bar plots are based on three independent biological replicates. Error bars indicate the standard deviation. In the qRT-PCR measurements, the values obtained were normalized to ACTB as a control. **(G)** WB assays using antibodies for ADAD2 and EIF2B4 in total cell lysates from 293T cells transiently transfected (24 hours) with a control plasmid (empty FRT) or one encoding the EBERs (FRT-EBER1/2). Also shown are the WB assays for PIK3AP1, ADAD2 and EIF2B4 in the BJAB-EBNA1-EBER1/2 *vs* BJAB-EBNA1 comparison. Error bars reflect the standard deviation of three biological replicates.

An unbiased GO analysis of the proteomics data cannot be done with the small number of proteins [24] exhibiting a significant fold-change in the SILAC experiment; typically at least 100 gene identification names are needed. Instead, inspection of the literature and GO databases determined that of the 8 upregulated proteins 6 (ADAD2, EIF2B4, FCRLA, FCRL1, PIK3AP1 and UBE2C) are associated with lymphocyte-specific gene expression or oncogenic signaling cascades. For the 12 downregulated proteins, no consensus in known biological functions emerged. From the 6 upregulated proteins mentioned above, we validated 5 (ADAD2, EIF2B4, FCRLA, FCRL1 and PIK3AP1) at the protein and/or mRNA levels by western blot (WB) and quantitative real-time polymerase chain reaction (qRT-PCR) assays, respectively, in control and EBER1/2-expressing BJAB cells (Fig 4).

ADAD2 (adenosine deaminase domain-containing protein 2) is an ADAR homologue with unknown function and activity [25] that could contribute to EBER1/2-specific RNA-editing events in B cells. Compared to the SILAC-based measurements, the upregulation of ADAD2 in BJAB-EBER1/2 cells measured by WB assays was subtle, but the values obtained were consistent across biological replicates (Fig 4A). At the mRNA level, the qRT-PCR data indicated low abundance of the mRNA and the *adad2* gene was not mapped in any of the mRNA-seq datasets collected (S5 and S9 Files). However, in support of our proteomics and WB data, ADAD2 was identified by MS/MS in B cells in the recently published draft of the human proteome [26]. As controls for the subtle fold-change observed, we confirmed by WB that the levels of the known EBER-interacting proteins La and L22, each identified in our proteomics dataset with a 1:1 SILAC ratio, did not change across biological replicates (Fig 4B).

The ADAR-mediated A-to-G editing pattern in mRNA transcripts has been studied in EBV-infected lymphocytes [27]. Prompted by the increase in ADAD2 protein levels, we assessed whether the EBERs, the most abundant EBV gene products, are responsible for the A-to-G editing patterns documented so far in B cells. The high coverage in our mRNA-seq datasets allowed us to show that EBER1/2 expression, whether at low (BJAB-EBER1/2 *vs* BJAB-CTL) or high (BJAB-EBNA1-EBER1/2 *vs* BJAB-EBNA1) levels, does not alter globally the A-to-G pattern compared to the corresponding BJAB control samples (S3A Fig). In accordance with this negative yet informative result, our SILAC data and the WB assays showed no changes in protein levels of the main A-to-G editing enzyme, ADAR1 (S3B Fig and S2 File).

The eukaryotic initiation factor 2 delta subunit (EIF2B4) is a component of the multiprotein GDP/GTP exchange factor EIF2B, which contributes to translation initiation by regenerating the EIF2/GTP complex [28]. This protein was interesting to validate since we do not observe a correlation between mRNA and protein levels in our cells, suggesting post-transcriptional regulation (Fig 3A and Table 1). EIF2B4 protein upregulation in BJAB-EBER1/2 cells measured by WB assays was subtle, as for ADAD2, but consistent across biological replicates. Unlike ADAD2, the WB measurements for EIF2B4 were similar to the SILAC ratio (Fig 4C). Both the mRNA-seq measurements (Table 1 and S3 File) and qRT-PCR data (Fig 4C) confirmed that the total *eif2b4* mRNA levels did not change upon EBER1/2 expression, suggesting post-transcriptional regulation. The upregulation of UBE2C (an ubiquitin-ligase that regulates the cell cycle) [29] suggests a possible post-translational regulation of EIF2B4 and UBE2C itself that like EIF2B4, showed no change at the mRNA level (Table 1).

To confirm that the upregulation of ADAD2 and EIF2B4 is EBER1/2-specific, we transfected the FRT plasmid used in the Flp-In strategy (Fig 1A), either empty or containing the two EBERs into 293T cells. We observed an increase close to 2-fold in the levels of both proteins (Fig 4G).

The FCR homologues (FCRL1-6 and FCRLA-B) are poorly characterized immunoglobulin-binding receptors [30]. The FCRLs are upregulated in centroblast lymphomas [31] and are normally expressed in germinal center B cells [32, 33], a required event for EBV latency stage II prior to the establishment of long-term latency I/0 [1, 2]. In our data, FCRLA and FCRL1 were included in one protein group (S2 File), even though they are distinct proteins encoded by different loci. Although we could not validate the upregulation of these proteins by WB due to poor antibody quality, we found that the total mRNA levels of both genes were upregulated in BJAB-EBER1/2 cells to a similar extent compared to their SILAC ratio (Fig 4D and Table 1). We also validated by qRT-PCR the total mRNA levels of the entire *fcrl* family expressed in human cells (Fig 4D), corroborating the mRNA-seq data Cuffdiff calculations (S3 and S4 Files). Given the documented expression of this family of genes in lymphomas [31], we asked if they were upregulated in the context of EBV infection. We found that the *fcrla* and *fcrlb* mRNAs increase significantly in BJAB-B1 cells, as to a lesser extent does *fcrl1* (Fig 4D *)*. Compared to any other Burkitt’s lymphoma EBV-positive cell line available, BJAB-B1 cells are near-isogenic to BJAB cells and thus expected to have a similar biochemical background. This is why we did not compare the EBER-expressing BJAB cells to other EBV-positive B cell lines. An example of this limitation is found in a recent paper in which gene expression data for BJAB and 293 cells expressing EBNA1 gave different results [34].

Finally, phosphoinositide 3-kinase adapter protein 1 (PIK3AP1), also known as B-cell adapter for phosphoinositide 3-kinase (BCAP), is a B-cell specific activator of the PI3K-AKT signaling cascade [35]. In the BJAB-EBER1/2 *vs* BJAB-CTL comparison, PIK3AP1 increased in protein level, as measured by WB, correlating with the SILAC ratio (Fig 4E). The total *pik3ap1* mRNA level determined by qRT-PCR increased slightly upon EBER1/2 expression (Fig 4E), confirming the subtle upregulation seen in the mRNA-seq calculations (Fig 3B and Table 1).

Prompted by this result, we also tested the predicted activation of AKT in BJAB cells and found that pAKT levels increase upon EBER1/2 expression (Fig 4F). Activation of the PI3K-AKT pathway is a well-characterized consequence of EBV latent infection [36] and is attributed specifically to the transmembrane EBV oncoproteins LMP1 and LMP2A [18]. We confirmed by WB that the levels of pAKT increase in BJAB-B1 cells, compared to the parental BJAB cell line (Fig 4F).

### High levels of EBER expression correlate with an oncogenic gene expression signature

To further corroborate our results and to more accurately mimic the physiological expression of EBER1 and EBER2 in EBV latency (high-level expression of EBERs), we compared the BJAB-EBNA1-EBER1/2 and BJAB-EBNA1 cells described above (Fig 1B) by mRNA-seq coupled to WB and qRT-PCR validation experiments. A SILAC proteomics experiment was not performed on these cells. In the BJAB-EBER1/2 *vs* BJAB-CTL comparison, many of the proteins encoded by genes up- or downregulated in the mRNA-seq data were not identified in the proteomics experiment (S2 File), most probably due to their low intracellular abundance and/or trypsin digestion patterns. While changes in total mRNA levels typically correlate with changes at the protein level [37], our observations of total mRNA level changes in the mRNA-seq data, coupled with WB validation assays, in most cases complement the partial proteome captured in the SILAC data we were able to obtain.

By WB of BJAB-EBNA1-EBER1/2 and BJAB-EBNA1 cells, we validated the ~2-fold upregulation of EIF2B4 protein, but not that of ADAD2 (Fig 4G). At the protein level, PIK3AP1 was also upregulated in these cells (Fig 4G). As expected, we also found that pAKT levels increase upon EBER1/2 expression in this system (Fig 4F).

**Fig 5:**
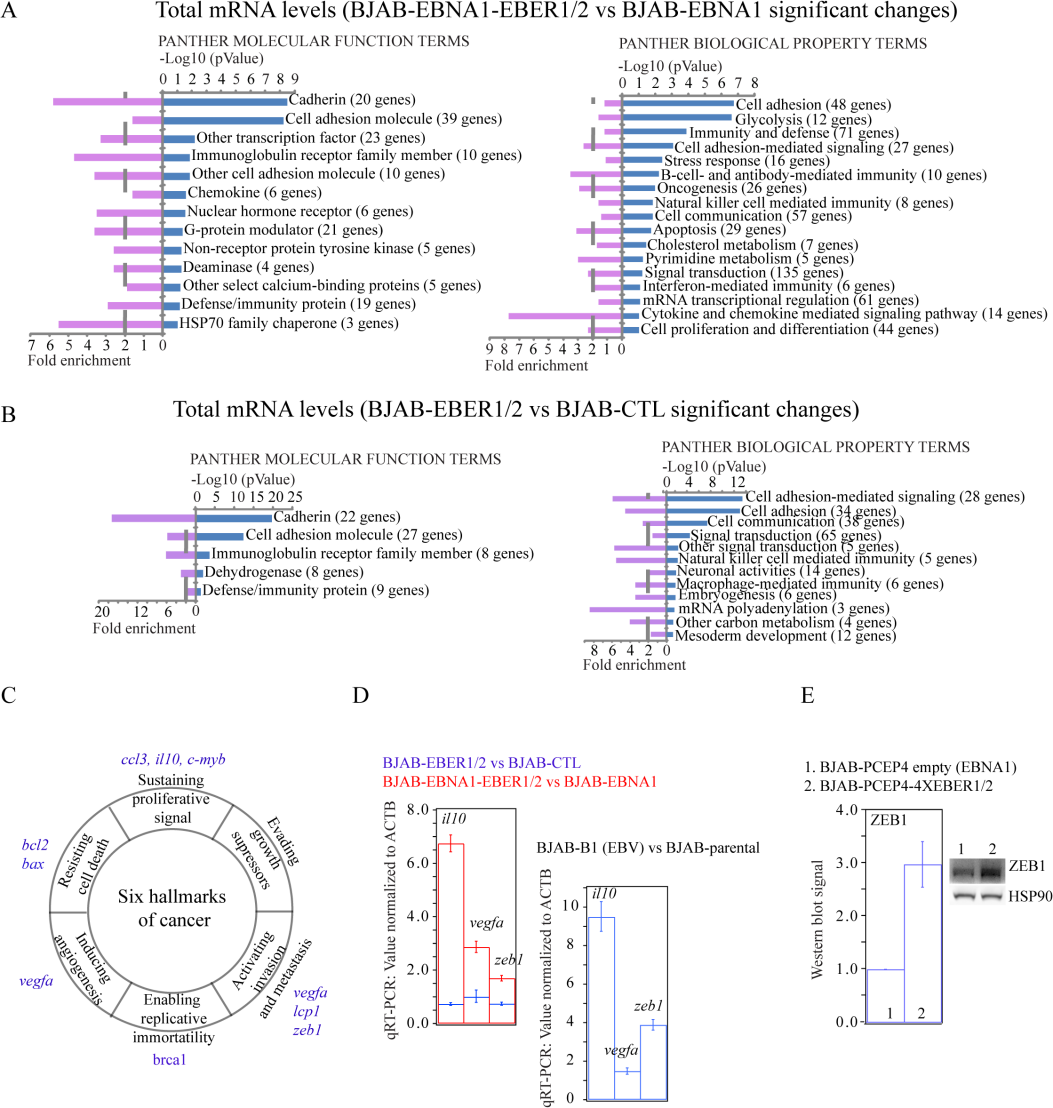
GO analysis and tumorigenic signature. Using the DAVID web-based portal (http://david.abcc.ncifcrf.gov/), we performed a GO analysis of the two datasets (low and high EBER1/2 expression) based on the main PANTHER classification terms, Molecular Function (MF) and Biological Property (BP). **(A)** GO analysis of the BJAB-EBER1/2 *vs* BJAB-CTL comparison (low EBER1/2 expression levels). Right and left plots indicate the enrichment in MF and BP terms, respectively. **(B)** As in the previous panel, GO analysis of the BJAB-EBNA1-EBER1/2 *vs* BJAB-EBNA1 comparison (high EBER1/2 levels). **(C)** Figure adapted from Hanahan and Weinberg [37] to highlight the oncogenic signature enriched in BJAB-EBNA1-EBER1/2 cells. **(D)** qRT-PCR validation assays for *il10*, *vegfa* and *zeb1* mRNAs from three biological replicates per comparison. The left plot shows an overlay of the BJAB-EBER1/2 *vs* BJAB-CTL (blue) and BJAB-EBNA1-EBER1-/2 *vs* BJAB-EBNA1 (red) fold-changes. The right plot shows the BJAB-B1 *vs* BJAB-parental fold-changes. All values were normalized to ACTB as a control. **(E)** WB assays with an antibody specific for ZEB1 in the BJAB-EBNA1-EBER1/2 *vs* BJAB-EBNA1 comparison. Densitometry values and error bars reflect three independent biological replicates.

To more thoroughly investigate the effects of high compared to low EBER expression, we interpreted the mRNA-seq data from BJAB-EBNA1-EBER1/2 and BJAB-EBNA1 cells. In the first replicate, Cuffdiff computed a total of 14,345 genes, of which 221 genes were upregulated (1.5%) and 506 downregulated (3.5%) significantly (S8 File). Compared to the 1.5% of upregulated genes in the first replicate of the BJAB-EBER1/2 *vs* BJAB-CTL comparison, the number of genes with a significant upregulation in the BJAB-EBNA1-EBER1/2 *vs* BJAB-EBNA1 dataset was similar even though their identity was not entirely the same (S9 File). A GO analysis of the genes changing significantly in total mRNA levels (according to their q-value) in the BJAB-EBNA1-EBER1/2 *vs* BJAB-EBNA1 comparison, revealed a tumorigenic gene expression signature (Fig 5A), absent in the data from BJAB cells that express low EBER levels (Fig 5B).

Our mRNA-seq data are not the first attempt to determine EBER-specific effects on mRNA levels in B cells. Previously, mRNA microarrays were used to investigate the effects on global mRNA levels in one of the two studies that failed to observe an obvious defect in EBER-minus EBV transformation of freshly-isolated lymphocytes (17). Mirroring the lack of obvious phenotype when compared to cells infected with an EBER-encoding EBV bacmid, the changes in mRNA levels reported in this study were subtle (17). We compared mRNA level changes from the two mRNA-seq datasets (low and high EBER expression) to the array data available (17) and failed to find commonalities (S3 File and supplementary files in [17]). This lack of commonalities is not completely unexpected because EBV-infected lymphocytes typically express the 9 EBV-encoded latent proteins, making them a common *ex-vivo* model system to study EBV latency III (3). In contrast, the BJAB cells used in our mRNA-seq comparisons expressing low (BJAB-EBER1/2) or high amounts of the EBERs plus EBNA1 (BJAB-EBNA1-EBER1/2) can be considered a surrogate of Latency 0 and Latency I, respectively.

At the isoform level, a total of 24,039 alternative isoforms were computed with confidence by Cuffdiff, of which 1433 (5.9%) changed significantly (S10 File). Tagged as significant, we found 285 (1.1%) alternative splicing events (S11 File), and 177 (0.7%) isoforms derived from alternative promoter usage (S12 File). These quantities were slightly lower, but still comparable to those in the BJAB-EBER1/2 *vs* BJAB-CTL comparison.

A GO analysis revealed a tumorigenic gene expression signature for the list of genes due to significant alternative splicing in the two datasets for low and high EBER expression (S4 and S5 Figs). As in the case of total mRNA levels, the list of genes with isoforms due to significant alternative promoter usage had an oncogenic signature only in the BJAB-EBNA1-EBER1/2 *vs* BJAB-EBNA1 comparison (S5 Fig).

To complement the proteomics profiles, we looked closer at the GO analysis performed on total mRNA level changes. In the list of genes classified in the GO term “Oncogenesis”, some had annotated functions populating five of the six so-called “hallmarks of cancer”, coined by Hanahan and Weinberg [38] (Fig 5C). We found that several of these oncogenes are associated with EBV-mediated lymphomagenesis. For example, *bcl2* is an antiapoptotic factor upregulated by the EBV protein LMP1 during latency [1, 2]; *ccl3* is a chemokine typically upregulated in lymphomas [39]; and *ccr7* is one of the first EBV-induced chemokine receptors documented [40]. Among other upregulated oncogenes is *il10*, known to increase in EBER-expressing cell lines [41, 42], plus *vegfa* and *zeb1*, both key components of metastatic progression [43, 44]. We validated the upregulated mRNA levels of *il10*, *vegfa* and *zeb1* in BJAB-EBNA1-EBER1/2 cells compared to BJAB-EBNA1 (Fig 5D) because these genes encode oncoproteins whose overexpression, somatic mutation and/or constitutive activity has been associated with oncogenic EBV latency [41, 42, 45]. In support of their tumorigenic role in lymphomas, these genes are also upregulated in BJAB-B1 cells (Fig 5D).

*Zeb1* encodes an E-box transcriptional repressor that is a key component of metastasis in cells of epithelial origin − its upregulation is a main trigger of the epithelial-to-mesenchymal transition (EMT) [44]. In the context of EBV infection of B cells, ZEB1 is a transcriptional repressor of the early EBV lytic gene *bzlf1/zebra*, whose protein product is important in the early stage of the lytic phase, during EBV DNA replication [46]. Given its functional relevance, we validated the increase in protein levels of ZEB1 in BJAB-EBNA1-EBER1/2 cells compared to BJAB-EBNA1 (Fig 5E).

**Fig 6:**
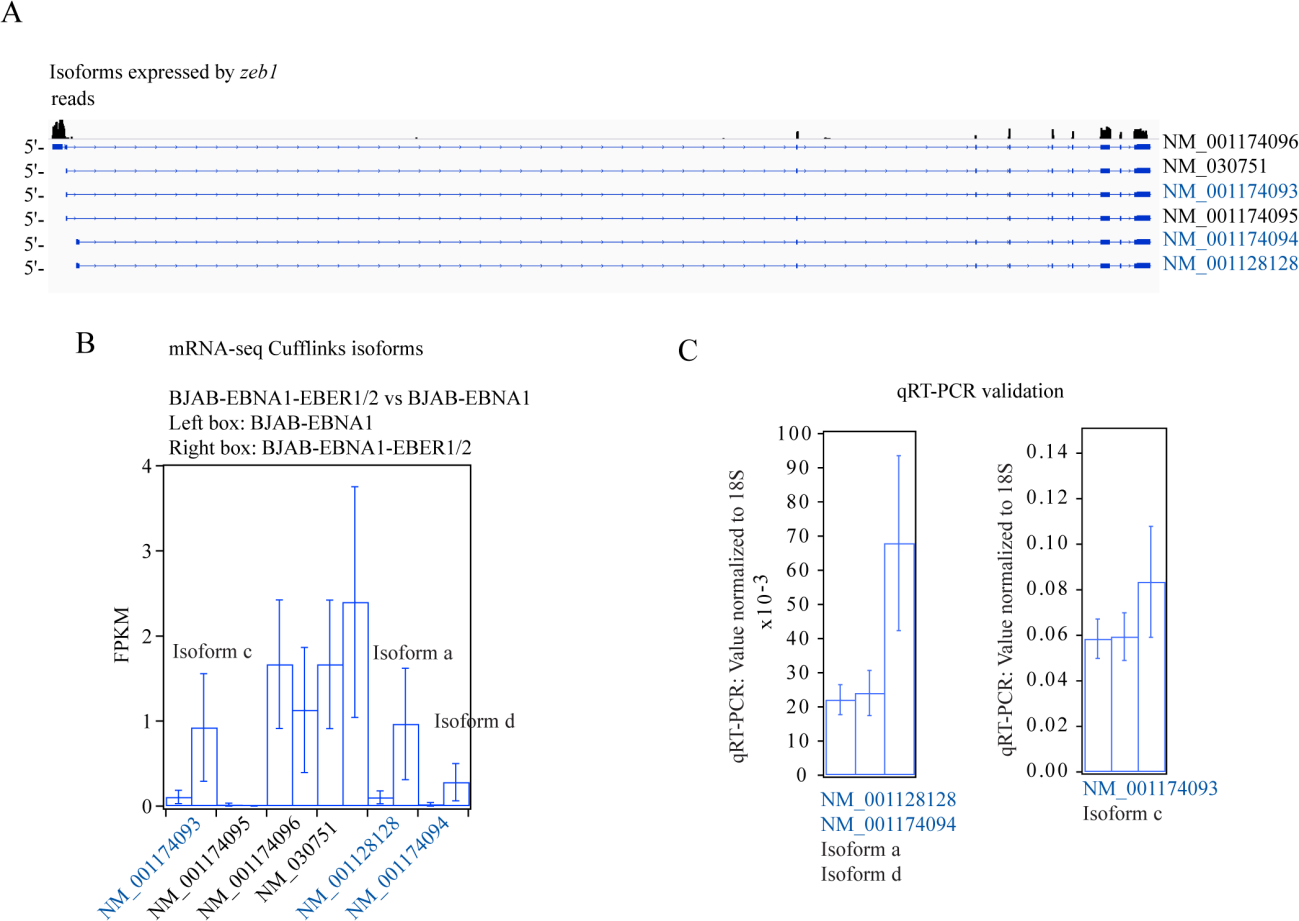
Validation by qRT-PCR of *zeb1* alternative isoform abundances in the BJAB-EBNA1-EBER1/2 *vs* BJAB-EBNA1 comparison. **(A)** The cartoon shows the mRNA-seq paired-end reads aligned by TopHat to the *zeb1* gene and indicates the alternative isoforms identified by Cufflinks in the bioinformatics analysis. The isoforms with a significant fold-change in the BJAB-EBNA1-EBER1/2 *vs* BJAB-EBNA1 comparison are colored blue. **(B)** Isoform abundances calculated by Cufflinks for each alternative isoform identified. The isoforms calculated to have changed significantly by Cufflinks are colored blue. **(C)** Validation by qRT-PCR of the indicated isoforms. The qRT-PCR values were normalized to 18S rRNA.

**Fig 7:**
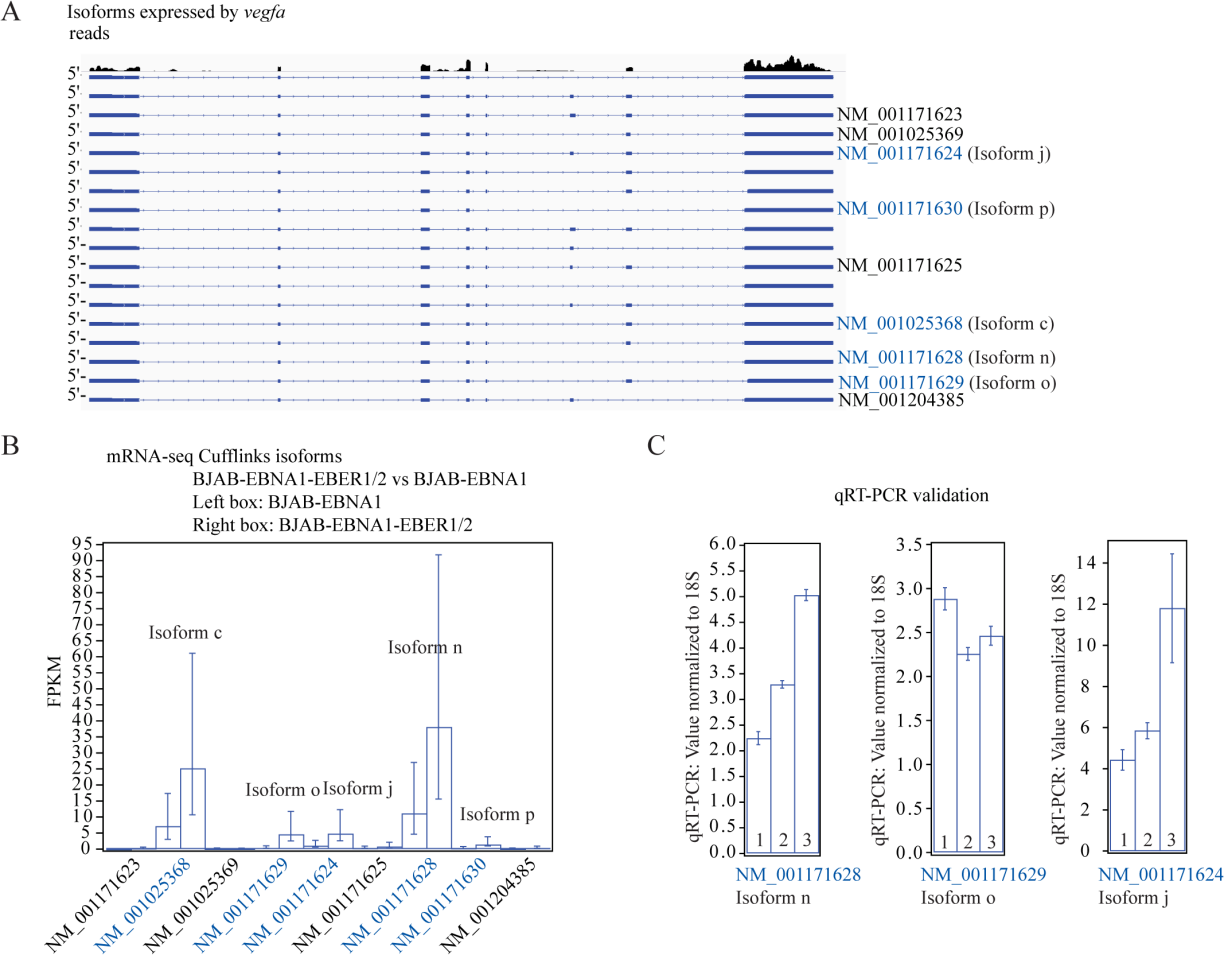
Validation by qRT-PCR of *vegfa* alternative isoform abundances in the BJAB-EBNA1-EBER1/2 *vs* BJAB-EBNA1 comparison. **(A)** The cartoon shows the mRNA-seq paired-end reads aligned by TopHat to the *vegfa* gene and indicates the alternative isoforms identified by Cufflinks in the bioinformatics analysis. The isoforms with a significant fold-change in the BJAB-EBNA1-EBER1/2 *vs* BJAB-EBNA1 comparison are colored blue. **(B)** Isoform abundances calculated by Cufflinks for each alternative isoform identified. The isoforms calculated to have changed significantly by Cufflinks are colored blue. **(C)** Validation by qRT-PCR of the indicated isoforms. The qRT-PCR values were normalized to 18S rRNA.

According to Cufflinks, the changes in *zeb1* individual isoform abundances were mainly due to alternative promoter-usage (Fig 6A and S10 and S12 Files). To demonstrate the accuracy of Cufflinks, we validated the changes in isoform abundance calculated for the gene *zeb1* (Fig 6A, 6B and 6C). We also validated the gene *vegfa* at the isoform level (Fig 7A, 7B and 7C). The *vegfa* gene has 19 alternative isoforms annotated in RefSeq and multiple alternative translation initiation sites [47] (Fig 7A). The complexity of this gene makes the analysis difficult for Cufflinks because the statistical power of this and other transcript-abundance computational tools decreases for genes with more than three isoforms [48]. Our mRNA-seq data indicated that only four of the 19 RefSeq-annotated isoforms were expressed at detectable levels, all upregulated with different fold-changes in the BJAB-EBNA1-EBER1/2 *vs* BJAB-EBNA1 comparison (Fig 7B). We designed specific primers for three of the four isoforms upregulated in the mRNA-seq data and were able to validate these by qRT-PCR assays (Fig 7C).

An unexpected result from the BJAB-EBNA1-EBER1/2 *vs* BJAB-EBNA1 comparison was the downregulation of *fcrla* and *fcrlb*, which we found to be upregulated in the SILAC data at low EBER expression (Fig 3A, 3B and 3D). These two genes were also upregulated in BJAB-B1 cells when compared to a control sample (BJAB parental) (Fig 4D). To investigate the occurrence of more cases in which the fold-changes had an opposite trend in the two datasets, we performed a pairwise comparison of the significant fold-changes at high EBER expression (which had an oncogenic GO term signature) and their corresponding values (whether the fold-change was significant or not, as indicated by the q-value) in the dataset at low EBER levels (S9 File). We found that only few fold-changes were simultaneously significant in the two datasets and that among these only a fraction had an opposite up- or downregulation trend, but of similar magnitude, like *fcrla* and *fcrlb* (S9 File). As expected, we found a similar trend at the isoform level (S9 File). These observations suggest that some genes, like *fcrla* and *fcrlb*, are up- or downregulated upon EBER-expression depending on the cellular context. An explanation may be the background of cellular activation induced by EBNA1 in addition to high EBER expression in the BJAB-EBNA1 *vs* BJAB-EBNA1-EBER1/2 comparison.

### The upregulated genes in both datasets are enriched in 3´-UTR AREs, but not in intronic AREs

A common feature of the upregulated oncogenes *bcl2*, *ccl3*, *ccr7*, *il10*, *vegfa* and *zeb1* is that their mRNAs contain AREs in their 3´-UTRs. Furthermore, *il10* and *vegfa* are well-documented substrates of AUF1, an ARE-binding protein that destabilizes ARE-containing transcripts [49, 50]. AUF1 interacts with EBER1, as we showed recently [10]. Thus, by sequestering AUF1, EBER1 might rescue ARE-containing mRNAs from degradation, causing an increase in their intracellular levels [10].

**Fig 8:**
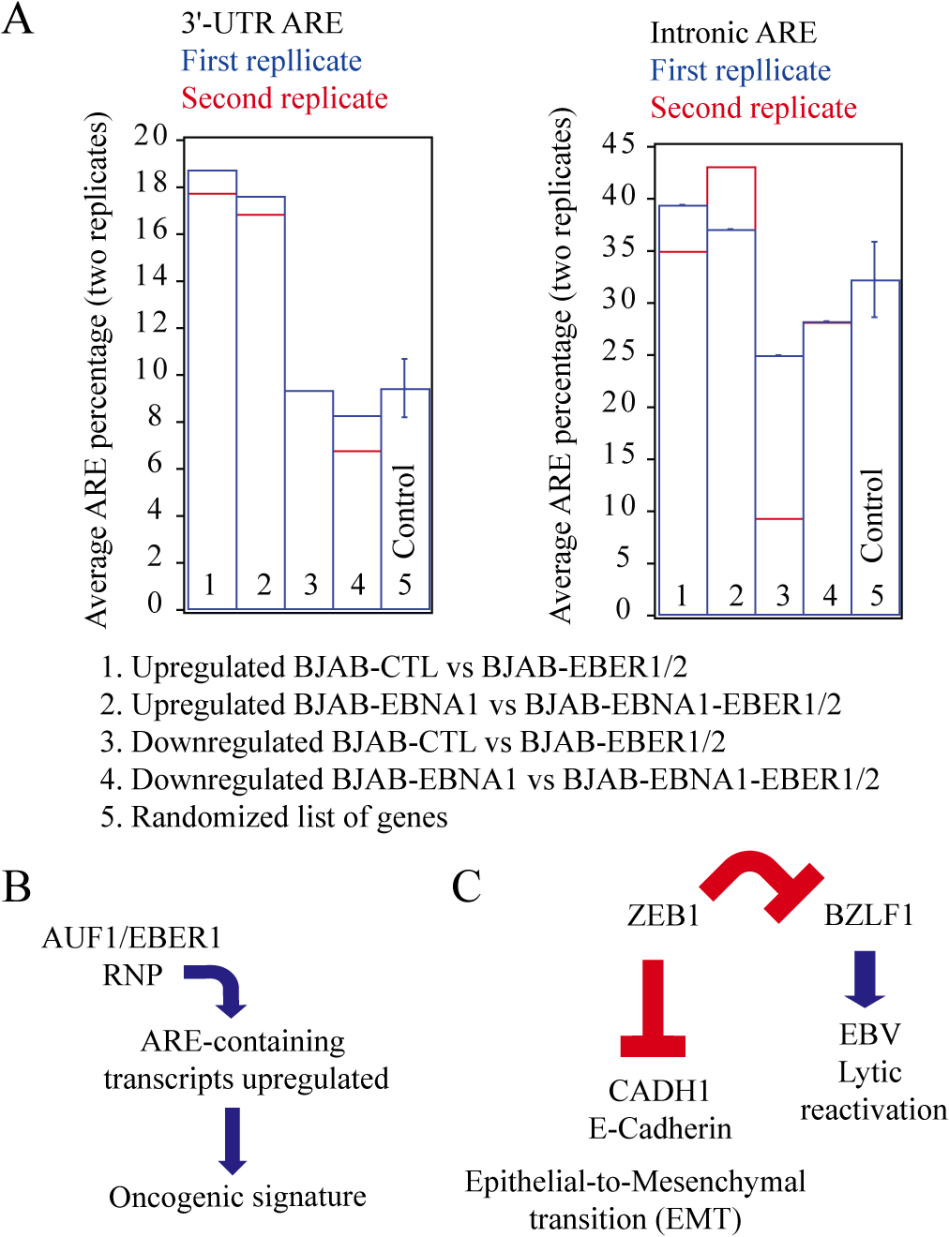
Enrichment of 3´-UTR AREs in the EBER-upregulated genes. **(A)** Enrichment of ARE-containing transcripts in the up- or downregulated list of genes in all datasets. We performed the analysis using the ARED repository of 3´-UTR or intronic AREs [45] by comparing the percentage in each list of genes with that generated by a randomly-picked list of 256 genes (performed 5 times). For each comparison (low and high EBER expression), we plotted the mRNAseq-derived data values as blue and red bars corresponding to the first and second biological replicates, respectively. In the case of the random list (control lane), the error bars reflect the standard deviation of the 5 random gene lists. No ARE-containing genes were found in the list of downregulated genes of the second biological replicate of the BJAB-EBER1/2 *vs* BJAB-CTL comparison. This explains the absence of a red bar plot in this case. **(B)** The mRNA-seq data support our previous conclusion that the ~1 million molecules of EBER1 per cell may prevent AUF1 from destabilizing 3´-UTR ARE-containing mRNA substrates–many of which encode oncoproteins [10]. **(C)** ZEB1 is a well-known master switch of the EMT, typically associated with metastasis [43]. This switch is used by EBV to maintain latency upon infection [42]. Our data suggest the model that EBER1 in high enough amounts may induce the upregulation of ZEB1, providing positive regulation of latency maintenance and in some cases oncogenesis.

We investigated the enrichment of ARE-containing genes in our data using the ARE database (ARED), which uses a trained set of experimentally verified ARE-containing motifs to identify the nonamer WWATTTAWW (W = A or T) in the 3´-UTR or introns of a transcript [51]. Among the up- and downregulated genes in the BJAB-EBER1/2 *vs* BJAB-CTL comparison, 18.75% of the upregulated and 9.37% of the downregulated, respectively, were annotated as ARE-containing (Fig 8A). Similarly, in the BJAB-EBNA1-EBER1/2 *vs* BJAB-EBNA1 dataset, 17.64% of the upregulated and 8.30% of the downregulated genes had one or more AREs in their 3´-UTRs (Fig 8A). We performed a similar analysis by choosing 254 genes (similar numbers of genes upregulated in the two mRNA-seq comparisons) at random and determining the ARE enrichment percentage. After five iterative repetitions, each with a different pool of randomly-chosen genes, we found an average enrichment of 9.44% for ARE-containing genes. This random enrichment is in accord with previous bioinformatic estimates that 7–10% of human genes have AREs in their 3´-UTRs [51]. As a control, we determined that the up- and downregulated total mRNA levels were not enriched in the recently annotated intronic ARE repeats (iARE) (Fig 8A).

## Discussion

We have combined SILAC proteomics and mRNA-seq transcriptomics to identify unique oncogenic signaling and gene expression features of an EBV-negative Burkitt’s lymphoma cell line (BJAB cells) that stably expresses the EBV ncRNAs, EBER1 and EBER2. We chose to work with BJAB cells in order to follow up our previous publication, in which using BJAB-B1 cells, we found that EBER1 interacts with AUF1 in a specific manner [10].

To identify unique effects of EBER expression, we first compared the proteome and mRNA transcriptome of BJAB-EBER1/2 *vs* BJAB-CTL cells (Figs 2 and 3 and Table 1). The levels of EBER1 and EBER2 in BJAB-EBER1/2 cells are ~10 times lower than in BJAB-B1 cells, infected with EBV (Fig 1A). We therefore corroborated the most relevant results obtained at low-level EBER expression in a comparison of BJAB-EBNA1-EBER1/2 *vs* BJAB-EBNA1 cells. BJAB-EBNA1-EBER1/2 cells express similar levels of EBER1 and EBER2, compared to BJAB-B1 cells (Fig 1B).

The SILAC data collected at low level EBER expression (BJAB-EBER1/2 *vs* BJAB-CTL) combined with WBs in the two comparisons (low- and high-level EBER expression) revealed that the B-cell specific AKT activator PIK3AP1 is upregulated in EBER-expressing B cells, along with an expected increase in pAKT (Fig 4E, 4F and 4G). The activation of AKT is known to affect on alternative splicing and translation [52]. Our data indicate post-transcriptional and and/or post-translational regulation of some genes, like EIF2B4 and UBE2C (Table 1 and Fig 4C). To our knowledge, EBER-mediated activation of AKT has not been documented previously even though the PI3K-AKT signaling cascade is a known target of the EBV oncoprotein LMP1 [18, 35] and a recent bioinformatics study showed that EBV infection affects alternative splicing [53]. This apparent redundancy in cell signaling activation mediated by EBER expression and the EBV-encoded oncoprotein LMP1 may explain the lack of effects on EBV transformation potential and latency maintenance observed in the EBER-deletion studies reported previously [16, 17].

To complement the proteomics profile, we collected mRNA-seq data at high EBER expression (BJAB-EBNA1-EBER1/2 *vs* BJAB-EBNA1) and focused on genes that exhibited fold-changes in total mRNA levels, for which, based on current studies, an equivalent change is expected at the protein level [36]. Compared to a random list of genes, we found that those with upregulated total mRNA levels are enriched ~2-fold in 3´-UTR AREs and that this enrichment is absent in those downregulated (Fig 8A). We observed this enrichment at low- and high-level EBER expression, but the ARE-containing upregulated genes in the BJAB-EBNA1-EBER1/2 *vs* BJAB-EBNA1 comparison populated an oncogenic gene expression signature, absent at low EBER expression (BJAB-EBER1/2 *vs* BJAB-CTL comparison). The increase in the levels of multiple ARE-containing mRNAs with annotated functions populating five of the six so-called “hallmarks of cancer” [37] (Fig 5C) may explain the contribution of EBERs to EBV-associated tumorigenesis.

These mRNA-seq results are novel because, unlike *il10* [40, 41], upregulation of the oncogenes *ccl3*, *ccr7*, *vegfa* and *zeb1* (Fig 5C) has to our knowledge not been reported previously in EBER-expressing cells. A study of EBV-mediated gastric carcinoma uncovered an increase in *zeb1* when an EBV-minus gastric cancer cell line was transfected with EBERs. This effect was not seen when the same cells were transfected with the EBV oncoproteins EBNA1 or LMP1 [54]. In accord with our previous conclusions [10], these results suggest that the ~10^6^ EBER1 molecules, present at all stages of EBV latency act to maintain the latent phenotype, possibly through their interaction with AUF1 and the consequent upregulation of ARE-containing genes (Fig 8B).

Previously, an explanation to the well-documented EBER-dependent upregulation of *il10* and other cytokines was the EBER-mediated activation of RIG-I, a cytoplasmic innate immunity sensor of microbial dsRNA [55]. This data however challenges the notion that EBER1 and EBER2 have been found to reside strictly in the nucleus [8], whereas RIG-I is a cytoplasmic protein [55]. Suggested by the mRNA-seq data in this paper and our previous finding that EBER1 interacts with AUF1, a mechanistic correlation between the specific interaction of AUF1 and EBER1 in the nucleus, better explains the upregulation of cytokines, such as *il10*.

Based on these results, our current model is that the upregulation of ZEB1 has a positive effect on viral latency maintenance and, alone or together with the increase of other 3´-UTR ARE-containing oncogenes, promotes cell proliferation, ultimately producing cancer (Fig 8C). Viruses of different types are known to negatively regulate host mRNA decay factors including AUF1 through a variety of mechanisms such as subcellular sequestration and proteolytic degradation [56]. While the validation of these results in other EBV-negative cell lines such as AKATA is necessary, this thorough proteomics and transcriptomics investigation of EBER-expressing BJAB cells therefore represents significant progress toward a mechanistic understanding of the role of EBERs in EBV latency.

## Supporting Information

S1 Fig SILAC proteomics strategy. **(A)** The scheme describes the protocol used in the SILAC proteomics experiment. To increase the number of proteins identified and their corresponding SILAC measurements, two independent sample preparations were performed, and each analyzed by LC-MS/MS in triplicate. SCX = strong cation exchange; RP = reversed-phase; LC = liquid chromatography; LTQ = linear triple quadrupole; and MS/MS = tandem mass spectrometry. ^12^C6 and ^13^C6 refer to the number of carbons in either Lys or Arg. The superscript indicates the isotope. **(B)** Histogram showing how the number of proteins identified is distributed in reference to their corresponding Log2 SILAC ratios. The data fit within a Gaussian curve. **(C)** Log10 of the total ion intensities per each protein identified plotted against their corresponding Log2 SILAC ratio as in panel **A**. **(D)** MS and MS/MS spectral quality of proteins identified with a significant SILAC ratio change and which were validated by WB and/or qRT-PCR assays.
(TIFF)

S2 Fig Northern blot assays of EBER1 and EBER2 in BJAB cells. **(A)** BJAB cells with the EBER1 or EBER2 gene integrated into a unique chromosomal site were generated by the Flp-In technology. As controls, we assessed EBERs in BJAB cells expressing one EBER. **(B)** BJAB cells stably transfected with the pCEP4 vector containing four, six or eight copies of the EcoRI-J fragment from the EBV genome compared to EBV-infected BJAB-B1 cells.
(TIF)

S3 Fig RNA-editing bioinformatics analysis and WB validation. **(A)** Using a stringent bioinformatics analysis protocol, we determined the instances in the genome in which an A-to-G change occurred (A-to-G event). We next calculated the relative A-to-G frequency per modified nucleotide, based on the number of reads allocated to the reference (A) and alternative (G) base. This histogram shows the degree of editing in each sample. **(B)** WBs for ADAR1 in the indicated comparisons.
(TIF)

S4 Fig GO analysis of the isoform changes in the BJAB-EBER1/2 *vs* BJAB-CTL comparison. The DAVID web-based portal (http://david.abcc.ncifcrf.gov/) was used to perform a GO analysis on the list of genes with a significant isoform switch event, explained by alternative splicing or promoter usage. **(A)** Alternative splicing. **(B)** Promoter usage.
(TIF)

S5 Fig GO analysis of the isoform changes in the BJAB-EBNA1-EBER1/2 *vs* BJAB-EBNA1 comparison. The DAVID web-based portal (http://david.abcc.ncifcrf.gov/) was used to perform a GO analysis of the BJAB-EBNA1-EBER1/2 *vs* BJAB-EBNA1 comparison. **(A)** Alternative splicing. **(B)** Promoter usage.
(TIF)

S1 File Supporting Information. This file contains the sequences of DNA primers and the source and identity of antibodies used in the manuscript. A detailed description of the bioinformatics analysis command lines used is also included.
(PDF)

S2 File SILAC. This file contains the complete SILAC dataset and a parsed list of proteins and their SILAC ratios alone or matched to their corresponding mRNA-seq ratio (average of two biological replicates). Values are in Log2.
(XLSX)

S3 File mRNAseq-replicates. This file contains the Log2 ratios of the two biological replicates in the mRNA-seq datasets, FRT (BJAB-EBER1/2 *vs* BJAB-CTL) and PCEP4 (BJAB-EBNA1-EBER1/2 *vs* BJAB-EBNA1).
(XLSX)

S4 File Gene-FRT. These files contain the changes in total mRNA levels in the FRT dataset, first and second biological replicates respectively. Plus the tracking output generated by Cuffdiff.
(XLSX)

S5 File Isoform-FRT. This file contains the changes in isoform levels in the FRT dataset, first and second biological replicates respectively. Plus the tracking output generated by Cuffdiff.
(XLSX)

S6 File Splicing-FRT. This file contains the complete dataset of the changes in alternative splicing usage in the FRT dataset.
(XLSX)

S7 File Promoter-FRT. This file contains the complete dataset of the changes in alternative promoter usage in the FRT dataset.
(XLSX)

S8 File Gene-PCEP4. This file contains the changes in total mRNA levels in the PCEP4 dataset, first and second biological replicates respectively. Plus the tracking output generated by Cuffdiff.
(XLSX)

S9 File mRNAseq-FRTvsPCEP4. This file contains the Log2 ratios of the significant total mRNAseq changes in the first biological replicate of the PCEP4 dataset (BJAB-EBNA1-EBER1/2 *vs* BJAB-EBNA1), matched to their corresponding values in the FRT dataset (BJAB-EBER1/2 *vs* BJAB-CTL).
(XLSX)

S10 File Isoform-PCEP4. This file contains the changes isoform levels in the PCEP4 dataset, first and second biological replicates respectively. Plus the tracking output generated by Cuffdiff.
(XLSX)

S11 File Splicing-PCEP4. This file contains the complete dataset of the changes in alternative splicing usage in the PCEP4 dataset.
(XLSX)

S12 File Promoter-PCEP4. This file contains the complete dataset of the changes in alternative promoter usage in the PCEP4 dataset.
(XLSX)
