# Supplementary figures and images for: Proteomics and Transcriptomics of BJAB Cells Expressing the Epstein-Barr Virus Noncoding RNAs EBER1 and EBER2
Source: PLoS One. 2015 Jun 29;10(6):e0124638. doi: 10.1371/journal.pone.0124638 (PMC4487896; doi:10.1371/journal.pone.0124638)

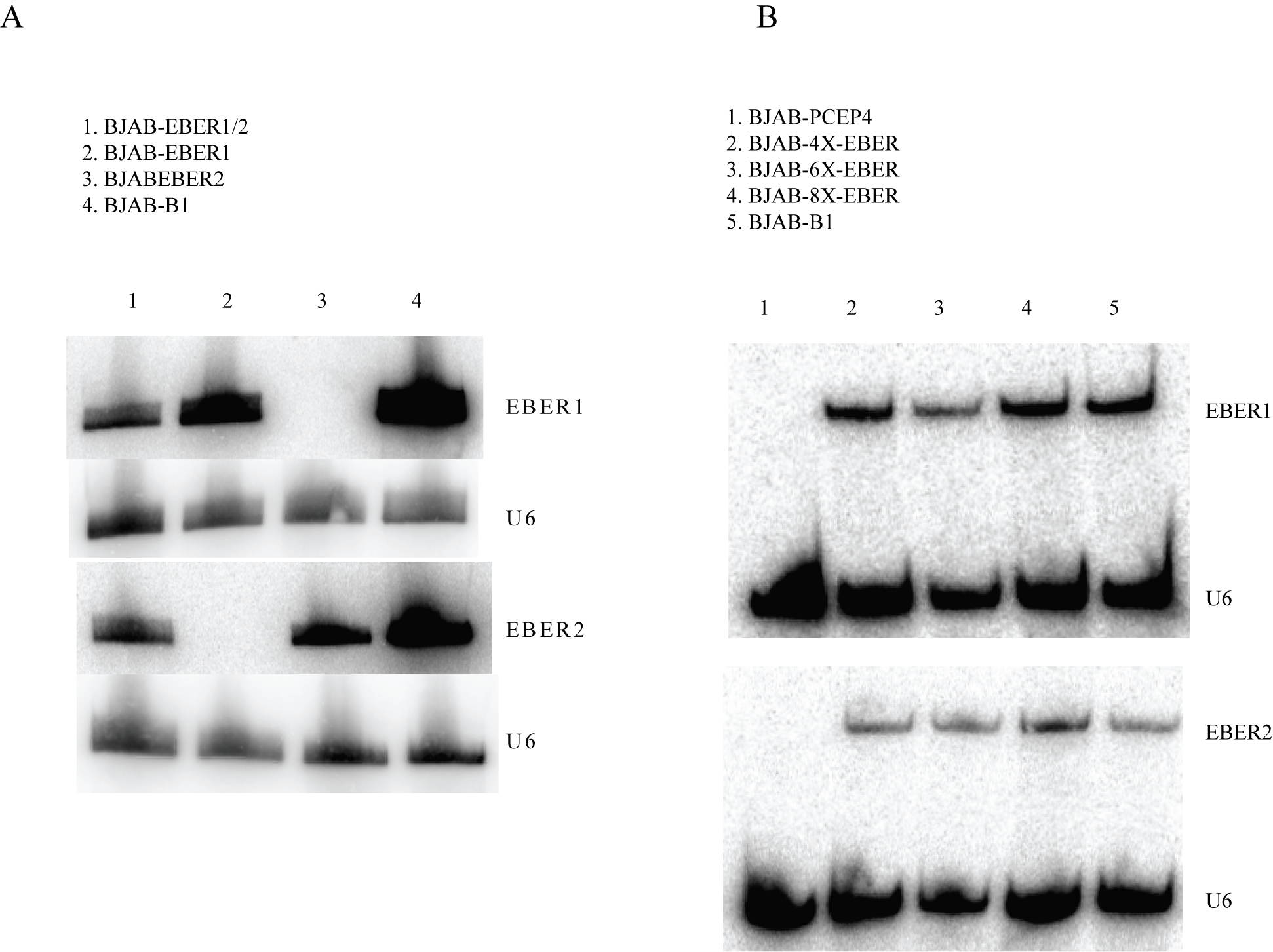

Supplement: S2 Fig — (A) BJAB cells with the EBER1 or EBER2 gene integrated into a unique chromosomal site were generated by the Flp-In technology. As controls, we assessed EBERs in BJAB cells expressing one EBER. (B) BJAB cells stably transfected with the pCEP4 vector containing four, six or eight copies of the EcoRI-J fragment from the EBV genome compared to EBV-infected BJAB-B1 cells. (TIF) [file pone.0124638.s002.tif]

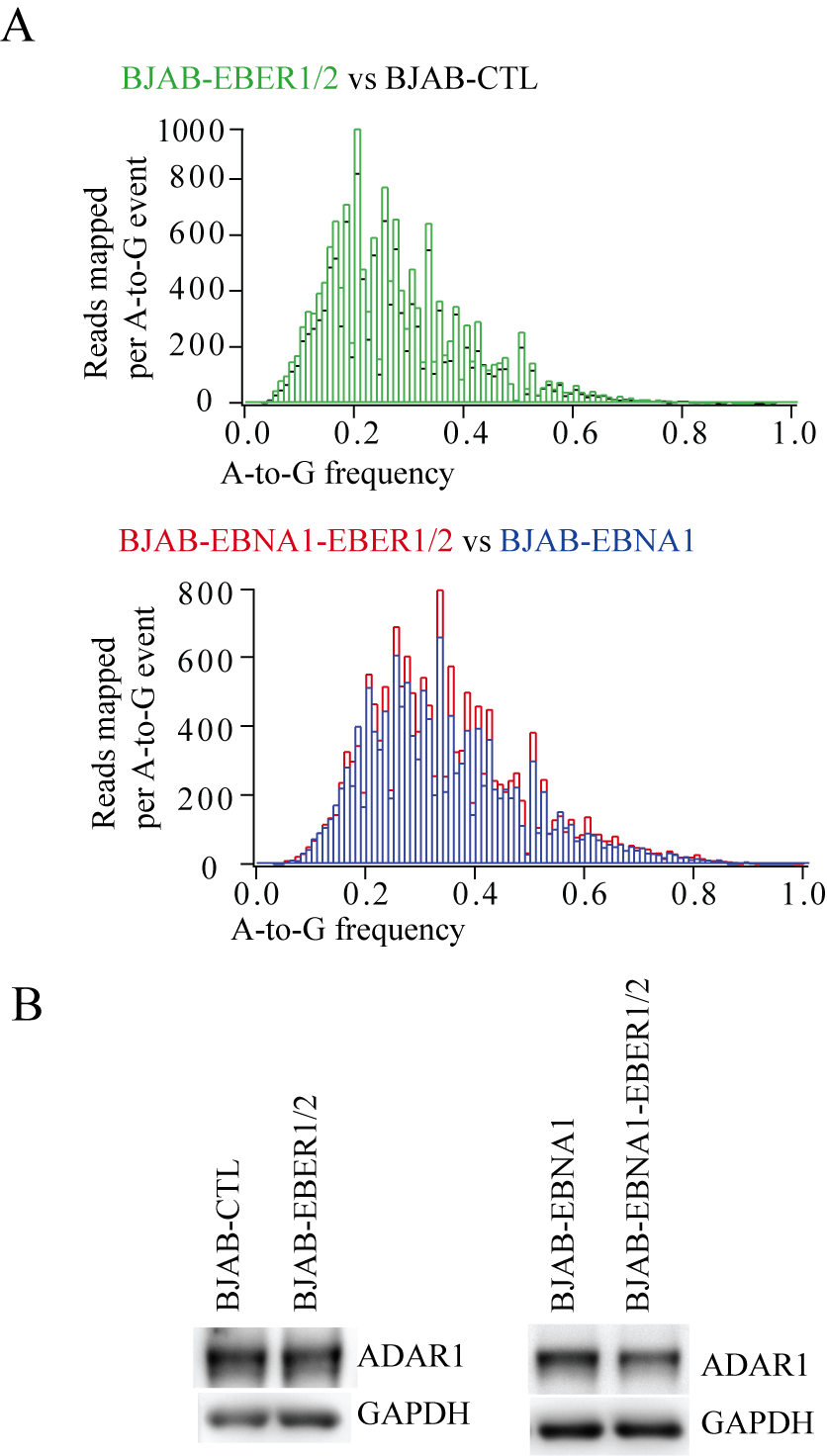

Supplement: S3 Fig — (A) Using a stringent bioinformatics analysis protocol, we determined the instances in the genome in which an A-to-G change occurred (A-to-G event). We next calculated the relative A-to-G frequency per modified nucleotide, based on the number of reads allocated to the reference (A) and alternative (G) base. This histogram shows the degree of editing in each sample. (B) WBs for ADAR1 in the indicated comparisons. (TIF) [file pone.0124638.s003.tif]

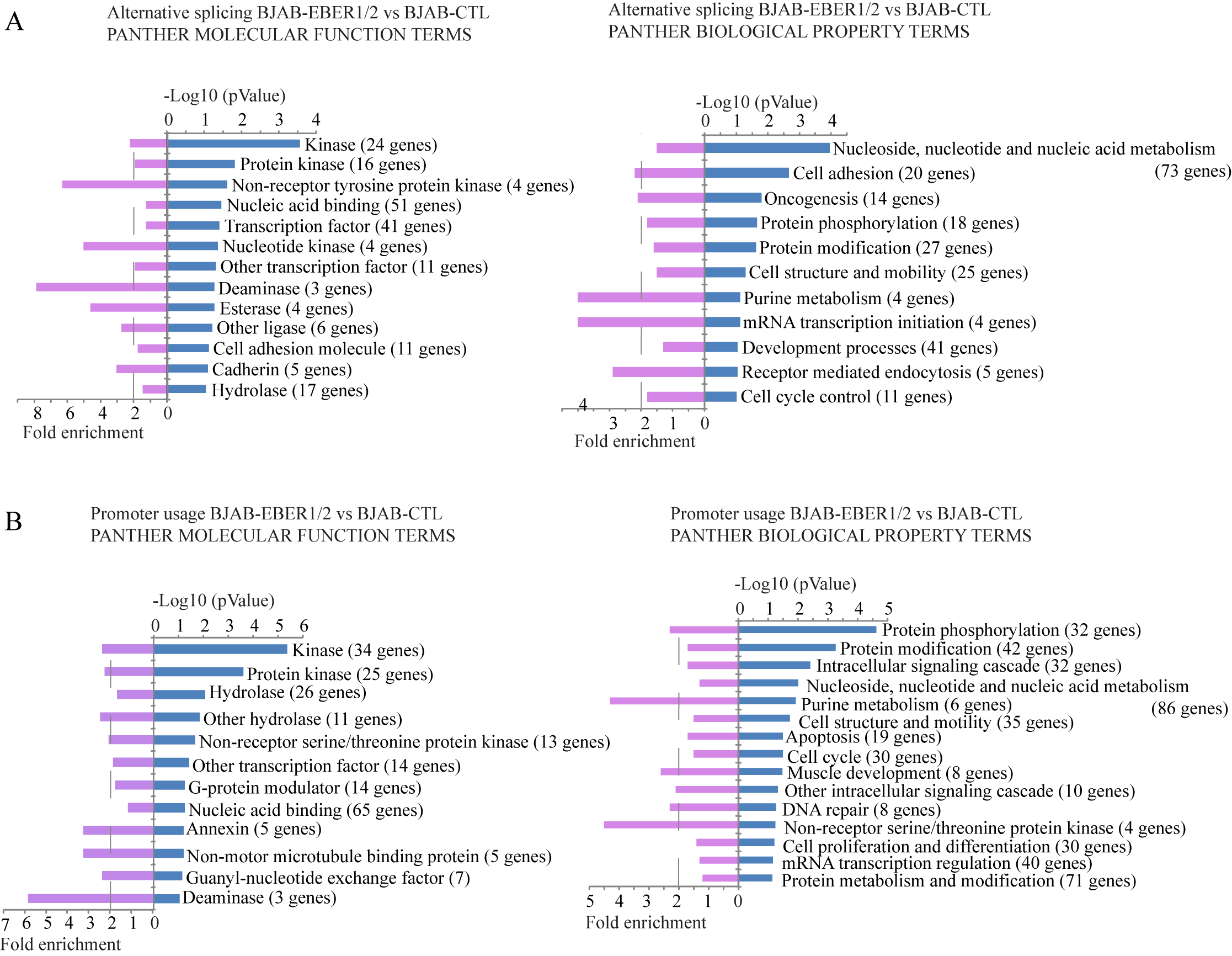

Supplement: S4 Fig — The DAVID web-based portal (http://david.abcc.ncifcrf.gov/) was used to perform a GO analysis on the list of genes with a significant isoform switch event, explained by alternative splicing or promoter usage. (A) Alternative splicing. (B) Promoter usage. (TIF) [file pone.0124638.s004.tif]

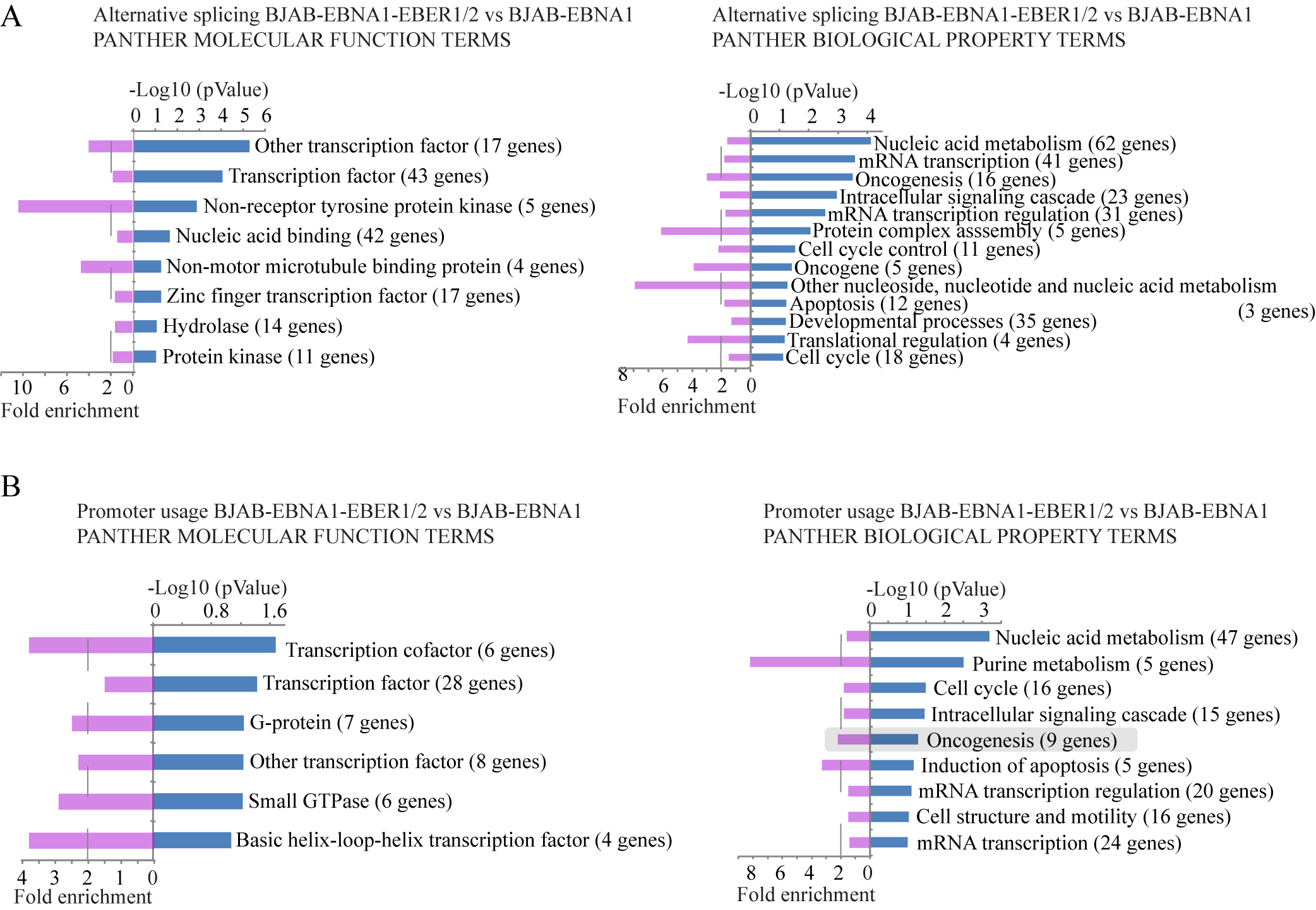

Supplement: S5 Fig — The DAVID web-based portal (http://david.abcc.ncifcrf.gov/) was used to perform a GO analysis of the BJAB-EBNA1-EBER1/2 vs BJAB-EBNA1 comparison. (A) Alternative splicing. (B) Promoter usage. (TIF) [file pone.0124638.s005.tif]
