# Supplementary material for: Proteomics and Transcriptomics of BJAB Cells Expressing the Epstein-Barr Virus Noncoding RNAs EBER1 and EBER2
Source: PLoS One. 2015 Jun 29;10(6):e0124638. doi: 10.1371/journal.pone.0124638 (PMC4487896; doi:10.1371/journal.pone.0124638)
Supplement: S1 File — This file contains the sequences of DNA primers and the source and identity of antibodies used in the manuscript. A detailed description of the bioinformatics analysis command lines used is also included. (PDF) [file pone.0124638.s006.pdf]

Supplementary information

All relevant data are within the paper and its Supporting Information files. Complete tables with the raw bioinformatics analysis outputs: mRNA-seq and SILAC proteomics calculations are included as supplementary files. We will provide an ftp link to whoever is interested in reanalyzing our data sets.

pCEP4 vector constructs

The primers used to amplify the EcoRI-J fragment from the pRIJ plasmid were:

5´-NheI

GTAGGACTAG gctagc CCA TAA AGC CTA GGG TGT AAA ACA CCG

3´-rev-BamHI

GTAGGACTAG ggatcc CCC TTT ACA TGT TGT GGG TGC AAA ACT

5´-BglII

GTAGGACTAG agatct CCA TAA AGC CTA GGG TGT AAA ACA CCG

3´-rev NheI

GTAGGACTAG gctagc CCC TTT ACA TGT TGT GGG TGC AAA ACT

qRT-PCR assays

Primers used:

Actb

Left primer CCTTGCACATGCCGGAG

Right primer ACAGAGCCTCGCCTTTG

Adar1

Left primer AGTCCTCTAGCTGTGGTAGG

Right primer CTCTGTTGCCAAGCTGGAGT

Ebna-1

Left primer: AGCGATAGAGCAGGGCCCCGCAGAT

Right primer: CAAAACCTCAGCAAATATATGAGTT

Eif2b4

Left primer GGAGGCTTGGACAGAGGG

Right primer GTGACATCACAGACCCGTTG

Fcrla

Left primer TGGAAGGATAGTGCAAAGCA

Right primer AGGAGCTGGATTCAATGTGG

Fcrlb

Left primer GTGTATCGATGCCAGACACG

Right primer AGACCACCTTGTCGTACCA

Fcrl1

Left primer TGATGAGCTGCTGCTGCT

Right primer AGGGCATCTTACACGTCAGG

Fcrl2

Left primer CCTTATGAAAATGCTTGAGGCT

Right primer CAGTGTCTGGATGTAGCAGCA

Fcrl3

Left primer AGTGAAGGGGTTTCCCATATG

Right primer GGCAGGAAGCTGCTACTCAG

Fcrl4

Left primer GCACAAATTTTGGTGAATTGG

Right primer CTGTGCAGGCAGAAGTGAAG

Fcrl5

Left primer CGCAGTGAGATGGTGACACT

Right primer GGGGGACGACCTATTTCCTA

Il10

Left primer CATGCTTCGAGATCTCCGAG

Right primer GCATCACCTCCTCCAGGTAA

Pik3ap1

Left primer GCGACATCCTCATCTACA

Right primer TGCTGAGGAAAAGGCTTAGG

Vegfa

Left primer CAACTTCTGGGCTGTTCTCG

Right primer GGCTGGAGCACTGTCTGC

Zeb1

Left primer CTGTGAAGGTGTACCAGAGGATGA

Right primer TTCTGCATCTGACTCGCATT

EBER1

Left primer AGGACCTACGCTGCCCTAGA

Right primer AAAACATGCGGACCACCAGC

SILAC bioinformatics data analysis

For data analysis, we used the software MaxQuant-Andromeda [1]. The parameters used were the same as in our previous publication [2]. As a matter of caution, we performed a manual inspection of the highest SILAC ratios with ≥ 2 counts and determined that the identification of

CPSF3 was not correct (despite its decent score) since the peptide sequenced was missing a trypsine-derived amino acid Arg or Lys (File S1-SILAC). We did not include this identification in our analysis.

Sample submission and data collection for mRNA-seq experiments

RNA samples were submitted to the Yale Center for Genomic Analysis (YCA) and their integrity was determined in a Bioanalyzer (Agilent). Only samples having an RNA integrity number (RIN) close to 10 (the highest score) were considered for library preparation. Staff at the YCA prepared cDNA libraries from mRNA (poly-A selected) using an in-house protocol.

A recent study in B cells determined that about 100 million reads (50bp, paired-end) are enough to capture confident measurements of more than 80% of total mRNA levels and 90% of individual transcript isoforms [3]. Furthermore, the initial publication describing Cufflinks made use of about 200 million reads, 75bp paired-end per sample [4].

Based on this study, we collected 75bp paired-end reads in HiSeq2000 and HiSeq2500 Illumina sequencers. In the first replicate, we collected 200-250 million paired-end reads. In the second replicate, we collected half that amount, approximately 100 million paired-end reads per sample. The sequencing data was preprocessed and made available by YCA's staff.

Bioinformatics transcript analysis using the Tuxedo suite of tools

To calculate gene expression levels, we used the Tuxedo bioinformatics suite − TopHat, Cufflinks and Cuffdiff tools [5]. To obtain information compatible with the proteomics data, we used the highly curated gene models provided by RefSeq to align reads, and assembled transcripts with Tuxedo. We used TopHat 2.0.08 to align paired-end reads guided by known gene models and the following command-line: tophat2 -g 1 -p 16 --no-novel-indels -G /genes.gtf -z pigz -o TopHat-output /Bowtie2Index/genome /left-reads.fastq /right-reads.fastq. The file

genes.gtf was the most recent RefSeq file with annotated gene models from the human genome. We used the latest genome hg19 in our analysis.

Next, we used Cufflinks 1.3.0 for transcript assembly with the following command-line: cufflinks -p 16 -v -u -b /genome.fa -G /genes.gtf -o Cufflinks-output TopHat-output.bam. For the quantitative comparison, we compiled the Cufflinks output files (replicates from different samples) with Cuffmerge 1.3.0, followed by Cuffdiff 1.3.0, using the following command-line: cuffdiff -p 16 -u -b /genome.fa -o Cuffdiff-output /Cuffmerge-merged.gtf /CTL-1st-replicate-TopHat-output.bam, /CTL-2nd-replicate-TopHat-output.bam /EBER-1st-replicate-TopHat-output.bam, /EBER-2nd-replicate-TopHat-output.bam.

As for total mRNA abundances, we used Cufflinks and Cuffdiff to obtain alternative isoform levels, using RefSeq gene models to align reads and assemble transcripts [5]. We first extracted the list of transcript isoforms up- and downregulated with a significant q-value. We then focused on relevant isoform switch events due to alternative splicing and promoter (significant Jensen-Shannon divergence metric, $\sqrt{JS}$ and the corresponding q-values), and that in most cases correlate to unchanged total mRNA levels.

Statistics of the alignments

We determined the quality of our datasets with the python script bamstat.py from the RNA-seq quality control suite of tools RSeQC [6].

The statistics were as follows:

BJAB-CTL first replicate

mapq >= mapq_cut (unique): 426642298

Read-1: 210982657

Read-2: 215659641

Reads map to '+': 213577511

Reads map to '-': 213064787

Non-splice reads: 307766108

Splice reads: 118876190

Reads mapped in proper pairs: 338398104

Proper-paired reads map to different chrom: 6218

BJAB-EBER1/2 first replicate

mapq >= mapq_cut (unique): 423658996

Read-1: 212291159

Read-2: 211367837

Reads map to '+': 212077843

Reads map to '-': 211581153

Non-splice reads: 302193094

Splice reads: 121465902

Reads mapped in proper pairs: 383188566

Proper-paired reads map to different chrom: 7190

BJAB-CTL second replicate

mapq >= mapq_cut (unique): 177833283

Read-1: 90619933

Read-2: 87213350

Reads map to '+': 89027972

Reads map to '-': 88805311

Non-splice reads: 133781987

Splice reads: 44051296

Reads mapped in proper pairs: 70859470

Proper-paired reads map to different chrom: 1810

BJAB-EBER1/2 second replicate

mapq >= mapq_cut (unique): 184885299

Read-1: 94127857

Read-2: 90757442

Reads map to '+': 92567915

Reads map to '-': 92317384

Non-splice reads: 140079661

Splice reads: 44805638

Reads mapped in proper pairs: 108012470

Proper-paired reads map to different chrom: 2368

BJAB-EBNA1-EBER1/2 first replicate

mapq >= mapq_cut (unique): 520711593

Read-1: 260338391

Read-2: 260373202

Reads map to '+': 260797994

Reads map to '-': 259913599

Non-splice reads: 377311579

Splice reads: 143400014

Reads mapped in proper pairs: 277509232

Proper-paired reads map to different chrom: 6618

BJAB-EBNA1 first replicate

mapq >= mapq_cut (unique): 535731196

Read-1: 268042586

Read-2: 267688610

Reads map to '+': 268328460

Reads map to '-': 267402736

Non-splice reads: 391772943

Splice reads: 143958253

Reads mapped in proper pairs: 304972136

Proper-paired reads map to different chrom: 7458

BJAB-EBNA1-EBER1/2 second replicate

mapq >= mapq_cut (unique): 117936279

Read-1: 59813905

Read-2: 58122374

Reads map to '+': 59016615

Reads map to '-': 58919664

Non-splice reads: 85389074

Splice reads: 32547205

Reads mapped in proper pairs: 16211722

Proper-paired reads map to different chrom: 756

BJAB-EBNA1 second replicate

mapq >= mapq_cut (unique): 114792438

Read-1: 58471765

Read-2: 56320673

Reads map to '+': 57424395

Reads map to '-': 57368043

Non-splice reads: 84155995

Splice reads: 30636443

Reads mapped in proper pairs: 8084824

Proper-paired reads map to different chrom: 768

Data parsing and gene ontology interpretation

To interpret our data, we manually parsed the Cuffdiff output files by converting them to Excel spreadsheets. We used simple scripts to make pair-wise file comparisons and plotted data with IgorPro 5.00. To interpret our data in terms of annotated gene/protein functions, we used the Database for Annotation, Visualization and Integrated Discovery (PORTAL) online source [7]. We searched a list of gene names up- or downregulated significantly according to Cuffdiff's q-value calculation, in DAVID using custom settings. In the DAVID portal, we focused on the output from the Protein Analysis Through Evolutionary Relationships (PANTHER) classification system [8]. We sorted the GO categories by fold-enrichment, and indicated their p-value. We also inspected the annotated functions of gene/protein names of interest in UniProt (http://www.uniprot.org/).

ADAR-mediated A-to-G analysis

To investigate the frequency of A-to-G events in our samples, we performed a bioinformatics analysis of each mRNA-seq dataset with a pipeline that included use of the following bioinformatics tools: BWA-0.6.2, SAMtools-0.1.18, Picard-Tools-1.72, GATK-2.4-3, BAMTools and BEDTools-2.17-0 [9-11].

The analysis pipeline, briefly described, was as follows:

1) Paired-end read alignment with Burrows-Wheeler Aligner (BWA).

2) Low-quality read end-trimming with CleanSam.jar (Picard-Tools).

3) Conversion of alignment files from the SAM (Sequence Alignment/Map) format to a binary version (BAM) with SAMtools.

4) Preparation of GATK (Genome Analysis Tools Kit) input files – processing each BAM file stepwise with the following Picard-Tools: SortSam.jar, FixMates.jar, MarkDuplicates.jar, AddReadGroups.jar and SortOrder.jar.

5) BAM file indexing with BAMTools.

6) "Variant calling" with Genome Analysis Tool Kit (GATK) following the "Best Practices" recommended workflow (http://www.broadinstitute.org/gatk/guide/best-practices).

The GATK pipeline was as follows: RealignerTargetCreator, IndelRealigner, BaseRecalibrator, PrintReads, ReduceReads, UnifiedGenotyper (multiple inputs simultaneously), VariantAnnotator, VariantRecalibator, ApplyRecalibration and SelectVariants.

The filters applied in the UnifiedGenotyper step were: -min_base_quality_score 25 -stand_call_conf 60 -stand_emit_conf 60 -dcov 100 -A QualByDepth -A FisherStrand -A DepthPerAlleleBySample -A HomopolymerRun.

7) Data parsing with BEDTools to remove known single nucleotide polymorphisms (SNPs), and extract A-to-G variants that occur in Alu repeats.

8) A-to-G frequency. Each base with a "variant call" had a number of reads per reference nucleotide (AD-REF) and alternative nucleotide (AD-ALT). We calculated the A-to-G frequency as AD-ALT/(ALT-REF + AD-ALT). We only considered AD-ALT values ≤ 2.

Western blots

The following antibodies were used:

Cell Signaling Technology: Phospho-Akt (Ser473) (193H12); Akt (9272); and ZEB1 (D80D3); and Abcam: ADAD2 (ab105737); eIF2B4 (ab96596); and PIK3AP1 (ab124031).

References

1. Cox J, Neuhauser N, Michalski A, Scheltema RA, Olsen JV, et al. (2011) Andromeda: a peptide search engine integrated into the MaxQuant environment. J Proteome Res 10: 1794-1805.
2. Lee N, Pimienta G, Steitz JA (2012) AUF1/hnRNP D is a novel protein partner of the EBER1 noncoding RNA of Epstein-Barr virus. RNA 18: 2073-2082.
3. Toung JM, Morley M, Li M, Cheung VG (2011) RNA-sequence analysis of human B-cells. Genome Res 21: 991-998.
4. Trapnell C, Roberts A, Goff L, Pertea G, Kim D, et al. (2012) Differential gene and transcript expression analysis of RNA-seq experiments with TopHat and Cufflinks. Nat Protoc 7: 562-578.
5. Trapnell C, Williams BA, Pertea G, Mortazavi A, Kwan G, et al. (2010) Transcript assembly and quantification by RNA-Seq reveals unannotated transcripts and isoform switching during cell differentiation. Nat Biotechnol 28: 511-515.
6. Wang L, Wang S, Li W (2012) RSeQC: quality control of RNA-seq experiments. Bioinformatics 28: 2184-2185.
7. Huang da W, Sherman BT, Lempicki RA (2009) Systematic and integrative analysis of large gene lists using DAVID bioinformatics resources. Nat Protoc 4: 44-57.

8. Mi H, Lazareva-Ulitsky B, Loo R, Kejariwal A, Vandergriff J, et al. (2005) The PANTHER database of protein families, subfamilies, functions and pathways. Nucleic Acids Res 33: D284-288.

9. Li H, Durbin R (2009) Fast and accurate short read alignment with Burrows-Wheeler transform. Bioinformatics 25: 1754-1760.

10. Li H, Handsaker B, Wysoker A, Fennell T, Ruan J, et al. (2009) The Sequence Alignment/Map format and SAMtools. Bioinformatics 25: 2078-2079.

11. McKenna A, Hanna M, Banks E, Sivachenko A, Cibulskis K, et al. (2012) The Genome Analysis Toolkit: a MapReduce framework for analyzing next-generation DNA sequencing data. Genome Res 20: 1297-1303.
